# Review of the ethnobotany, phytochemistry, pharmacology, and toxicity studies of the genus *Adenia*

**DOI:** 10.3389/fphar.2025.1581659

**Published:** 2025-05-21

**Authors:** Iliassou L. Mouafon, David R. Katerere

**Affiliations:** Department of Pharmaceutical Sciences, Tshwane University of Technology, Pretoria, South Africa

**Keywords:** *Adenia*, ethnobotany, bioactive compounds, pharmacology, ribosome-inactivating proteins, toxicity, traditional medicine

## Abstract

**Background:**

The genus *Adenia* has a rich history of traditional medicinal use across various cultures, particularly in Africa and parts of Asia. The present review summarizes key features of the genus *Adenia*, focusing on its occurrence, distribution, isolation, bioactivities, and toxicities.

**Material and methods:**

A thorough literature review was conducted using databases such as Google Scholar, PubMed, ScienceDirect, JSTOR, and Web of Science. The search utilized the keyword “*Adenia*” in combination with relevant terms like “distribution,” “traditional use,” “phytochemicals,” “chemical compounds,” “pharmacology,” “bioactivity,” and “toxicity.”

**Results:**

Our search yielded 3,724 records, with 88 studies from 1935 to 2024 meeting our criteria. The findings indicate that the genus *Adenia* comprises over 106 species of climbing plants, commonly found in tropical and subtropical regions. In traditional medicine, several *Adenia* species have been employed in various cultures for medicinal purposes, treating ailments such as wounds, leprosy, malaria, infections, infertility, colic, dysentery, hypertension, rheumatism, headaches, abdominal pain, and cancer. Chemical investigations have identified 27 secondary metabolites including alkaloids, glycosides, flavonoids, and ribosome-inactivating proteins (RIPs), particularly type 2, which are associated with cytotoxic and toxic effects. Pharmacological studies of chemical constituents and extracts from *Adenia* species have revealed a broad spectrum of biological activities, including cytotoxicity, antimicrobial, antioxidant, anti-inflammatory, analgesic, anticholinesterase, neuropharmacological, antidepressant, antihyperglycaemic, anti-anemic, anticoagulant, antithrombotic, thrombolytic, and anesthetic activities. Despite their therapeutic benefits, concerns regarding safety and toxicity are significant, necessitating comprehensive evaluations and standardized methodologies for assessing their efficacy. Thus, future research should focus on validating the traditional uses of *Adenia* species through rigorous scientific methods to ensure their safety and efficacy in modern medicine.

## 1 Introduction

The genus *Adenia* Forssk. (Passifloraceae) comprises over 106 species of climbing plants, predominantly found in tropical and subtropical regions of Asia, Africa, and the Pacific Islands. This genus is notable for its extensive morphological diversity and ecological versatility, allowing species to thrive in various environments, from arid landscapes to lush rainforests (Hearn, 2007; Sinei and Mwangi, 2015; Rotich, 2022). Traditionally, *Adenia* species are utilized across numerous cultures for medicinal purposes, with different parts of the plant—such as leaves, roots, stems, fruits, and seeds—being employed to treat various ailments, including wounds, fever, gastrointestinal issues, pain, and inflammation (Sarkodie et al., 2013; Sinei and Mwangi, 2015; Barua et al., 2020; Maroyi, 2020; Rotich, 2022; Chibuye et al., 2024). The presence of bioactive compounds further underscores the therapeutic potential of these species.

Ethnobotanical studies have consistently emphasized the importance of *Adenia* species in traditional medicine, particularly in Africa, where species like *A. gummifera*, *A. cissampeloides*, and *A. lobata* are utilized for various medicinal applications (Ibiam et al., 2015; Ogunlesi et al., 2019; Garba Koffi et al., 2021; N’Go et al., 2021; Rotich et al., 2021; Florian et al., 2022). The pharmacological relevance of these plants is enhanced by their chemical diversity, featuring numerous secondary metabolites including alkaloids, glycosides, flavonoids, steroids, and terpenoids (Ogunlesi et al., 2019; Garba Koffi et al., 2021; Immaculate et al., 2022). These compounds are believed to contribute to the pharmacological effects seen in *Adenia* species, including antimicrobial, antioxidant, anticancer, anti-inflammatory, and analgesic activities (Fowa et al., 2019; Barua et al., 2020; Maroyi, 2020; Bortolotti et al., 2021; N’Go et al., 2021; Rotich, 2022). Notably, ribosome-inactivating proteins (RIPs), particularly type 2, may play a significant role in the cytotoxic and toxic properties of certain *Adenia* species, with several demonstrating selective toxicity against cancer cell lines, making them promising candidates for anticancer drug development (Tosi et al., 2009; Battelli et al., 2010; Bortolotti et al., 2021; Maiello et al., 2021; Rotich, 2022).

Despite their potential therapeutic benefits, concerns about the safety and toxicity of *Adenia* species are growing, especially given their extensive use in traditional medicine. Limited toxicological data indicate that some species may exhibit adverse effects at specific dosages (Ekpo et al., 2017; Uno et al., 2018; Barua et al., 2020; Rage et al., 2024). This highlights the necessity for thorough safety evaluations before recommending *Adenia* products for therapeutic purposes. Furthermore, the lack of clinical data regarding the safety, efficacy, and pharmacokinetics of *Adenia* compounds necessitates more rigorous research to establish clear guidelines for their medicinal use. This review aims to synthesize the existing literature on the ethnobotany, phytochemistry, pharmacology, and toxicity of the genus *Adenia*. By exploring traditional uses, chemical constituents, pharmacological properties, and potential risks, this review underscores the therapeutic potential and the need for further scientific investigation into these remarkable plants.

## 2 Methodology

A thorough literature review was carried out using databases like Google Scholar, ScienceDirect, PubMed, JSTOR, and Web of Science, covering articles published from 1935 to 2024. These databases were selected for their relevance to medicinal plant research and their recognition by peer-reviewed literature. The search utilized the keyword “*Adenia*” combined with terms like “distribution,” “traditional use,” “phytochemicals,” “chemical compounds,” “pharmacology,” “bioactivity,” and “toxicity.” For botanical descriptions, the Plant List was accessed via WFO plant list, to compile an exhaustive list of all published *Adenia* species. Articles were evaluated based on their titles and abstracts, with the following inclusion criteria: (1) only full-text articles or reports that were published in or translated into English; (2) original peer-reviewed studies or reports focusing on the ethnobotany, pharmacology, phytochemistry, and toxicity of *Adenia* species. Exclusion criteria included papers that (1) did not study *Adenia* species; (2) were not full-text articles; and (3) were narrative or systematic reviews, or reports lacking original data (such as editorials, expert opinions, and perspective papers). To avoid duplicate results, all downloaded articles were imported into EndNote^®^ X9 (Thomson Reuters, Philadelphia, PA, USA), where duplicate reports were removed.

## 3 Results and discussion

Our electronic database search generated 3,724 records. After deduplication, screening titles and abstracts, and applying our inclusion and exclusion criteria, 88 studies from 1935 to 2024 were selected for full-text review. The evidence from these included studies is summarized narratively below and listed in Table 1. The review begins with an overview of the botanical description and distribution of *Adenia* species, followed by sections on their ethnobotany and medicinal uses, phytochemistry, pharmacology, and toxicity studies.

**TABLE 1:** Ethnomedicinal importance of the species from the genus *Adenia*.

Name of the plants	Geographical location	Vernacular name (local dialect, Country)	Parts used	Recipes preparation	Administration	Traditional uses	References
*A. gummifera* var. gummifera	South Africa, the Democratic Republic of Congo, Ethiopia, Eswatini, Kenya, Mozambique, Malawi, Seychelles, Somalia, Uganda, Tanzania, Zimbabwe, and Zambia	Snake climber (English) kinyelwet (Kipsigis, Kenya) Imfulwa (Zulu, South Africa) Muhore (Shona, Zimbabwe) Mandali (Suaheli)	Root, Leaf, whole plant	Infusions Decoction Pounding Crushing	Oral tropical	Depression, respiratory infections, sexually transmitted infections, infertility, leprosy, malaria, lethargic birds, flu-like symptoms, coughing, coccidiosis, blindness, colic while and broken bones. Used as emetics, disinfectant or diuretic	Adedapo et al., 2008; Fullas et al., 1995; Jambwa et al., 2022; Luman and Mottram, 2006; Maroyi, 2020; Rotich et al., 2021; Rotich, 2022
*A. cissampeloides* (Planch. ex Hook.) Harms	West Africa, distributed from Senegal to Somalia, with a notable presence in Ghana and Nigeria	Monkey rope (English) Muore (Zimbabwe) Arokeke or Godogbo (Yoruba, Nigeria)	Root, Stem, Leaf	Infusions Decoction Maceration Crushing Shewing Pounding	Oral Tropical	Malaria, fever, dysentery, rheumatic pain, hypertension, numbness, edema, constipation, diarrhea, wound dressing, headaches, back pain, leprosy, cholera, anemia, liver issues, intestinal worms wounds, sores broken bones, fractures, depression and insanity. Used as emetic and diuretic	Agbor and Okoi, 2014; Annan et al., 2012; Ekpo et al., 2017 Ibiam et al., 2015; Ishola et al., 2015; Neuwinger, 2004; Ngarivhume et al., 2015; Nyansah et al., 2016; Ogunlesi et al., 2019; Uno et al., 2018; Uzoeto et al., 2018
*A. lobata* (Jacq.) Engl.	It extends from Senegal to Angola and Cameroon, particularly in Côte d'Ivoire, Ghana, and eastern Nigeria.		Not specified	Not specified	Oral	Jaundice, headaches, abdominal pains, otitis, malaria, infantile asthma, coughs, rheumatic, respiratory disorders, syphilis, gonorrhea, nose cancer anemia gingival inflammation, and diabetes mellitus.	Agoreyo et al., 2012; Bertrand Fowa et al., 2019; Florian et al., 2022; Fowa et al., 2019; Garba Koffi et al., 2021, Koffi Marcel et al., 2011; N’Go et al., 2021; Sarkodie et al., 2013
*A. trilobata* (Roxb.) Engl.	Chittagong district in Bangladesh, Assam, Pakistan, Myanmar, and the western and eastern Himalayas	Akandaphal (Bangladesh)	Leaf	Concoction Poultice	Oral Tropical	Diabetes, arthritis, and toxicity, headaches, stomach issues, knee pain, and snake bites	Aziz et al., 2020; Barua et al., 2020; Islam et al., 2022
*A. glauca* Schinz	Dry bushveld of southern Africa	Elephant foot (English)	Not specified	Powder	Tropical	Ear infections, swollen legs, and skin diseases.	Immaculate et al., 2022, 2023; Spencer and Seigler, 1982a
*A. volkensii* Harms	Kenya	Kiliambiti (Kamba, Kenya)	Not specified	Not specified	Oral	Poison to kill hyenas	Barbieri et al., 1984; Gondwe et al., 1978
*A. digitata* (Harv.) Engl.	South Africa	Modecca (South Africa)	Not specified	Not specified	Oral	Poison	Spencer and Seigler (1982b)
*A. panduriformis* Engl.	Zambia, Mozambique, Tanzania, and Zimbabwe	Not Found	Leaf	Not specified	Oral	Degenerative conditions, diabetes, hypertension, malaria, diarrhea, and immune booster for patients with HIV/AIDS	Chibuye et al. (2024)
*A. wightiana* (Wall. ex Wight and Arn.) Engl.	Not found	Not found	Leaf	Not specified	Oral	Peptic ulcers.	Presena and Pragasam (2016)
*A. viridiflora* Craib	Not Found	Not Found	leaf, flower, fruit, and shoot	Infusions Decoction Maceration	Oral	Fever, urinary tract infection, giddiness, diarrhea, fainting, obesity, diabetes, hypertension, and Alzheimer’s disease	Wannasaksri et al. (2021a)
*A. globosa* Engl.	Kenya	Not found	Tuber	Juice Infusion	Oral	Facilitate and expedite parturition on domestical animals experiencing difficulties during delivery.	Gruber and OʼBrien, 2011; Sinei and Mwangi, 1995; Sinei, and Mwangi, 2015

### 3.1 Botanical description and distribution

The genus *Adenia*, a group of flowering plants in the family Passifloraceae, is characterized by its climbing or sprawling habit, with stems emerging from above-ground tubers that can reach up to 2.5 m wide and feature fleshy, lobed leaves (Liebenberg, 1935; Sinei and Mwangi, 2015). It comprises around 106 species and is considered the second-largest genus in the family after *Passiflora* with over 500 species (Rotich, 2022). The genus *Adenia* is primarily found in the Old World tropics, where it has undergone significant evolutionary diversification. Its plants are adapted to diverse environments, including dry and moist forests, savanna woodlands, and bushlands (Sinei and Mwangi, 2015). It shows a notable diversity in growth forms, ranging from succulent trees, shrubs, and vines to geophytes, lianas, and other unique growth habits. It is believed that the ancestral *Adenia* species was likely a liana with storage roots (Hearn, 2006). Several species within *Adenia* have evolved distinct traits, such as stem succulence, which is thought to have evolved independently at least three times, and the development of storage roots, with multiple gains and losses of these features across different species (Hearn, 2006; Hearn, 2009). Regarding morphological variation, *Adenia* species can range from small herbaceous climbers to larger, woodier plants (De Wilde, 1971). Many species are dioecious, meaning that male and female flowers are found on separate plants, though some species exhibit monoecy. The flowers, generally glabrous, have variable shapes, with the male flowers being smaller than the female flowers. The fruits of *Adenia* are typically 1-celled capsules that dehisce loculicidally, leaving seeds attached to the middle of the valves. Fruit shape can range from fusiform to globular, with colors varying from greenish to yellow or bright red when ripe (De Wilde, 1971).

**FIGURE 1:**
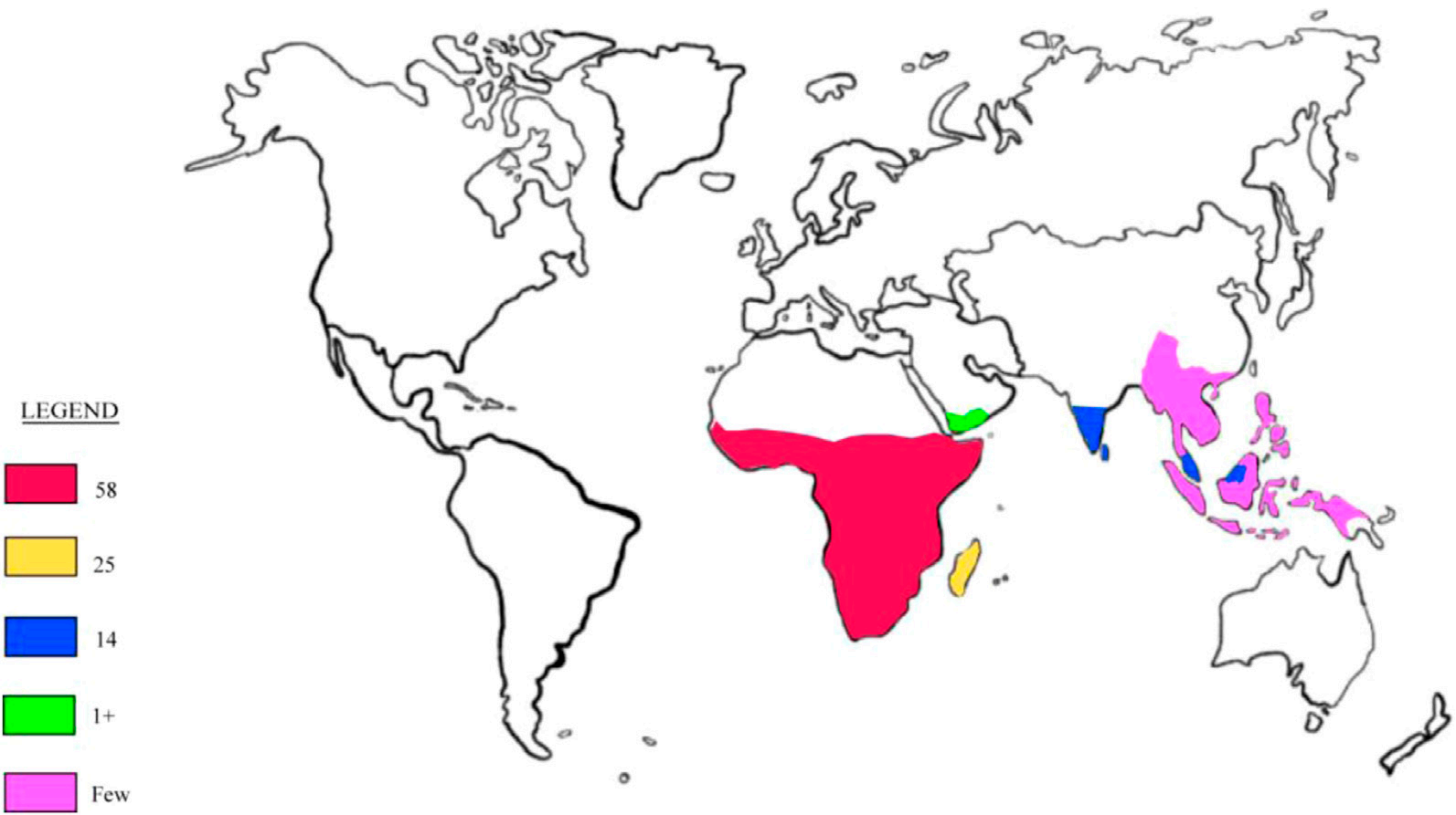
Global distribution of the genus *Adenia*.

The distribution of *Adenia* (Figure 1) is predominantly paleotropical, with a significant concentration in Africa, where 58 species are found, alongside 25 species in Madagascar and 14 species in South India, Ceylon, and Indo-Malesia (De Wilde, 1971; Hearn, 2007). The genus extends as far north as the savanna belt south of the Sahara, passing through Sudan and Ethiopia, and into Yemen, where *A. Venenata,* the first species described, is found (Liebenberg, 1935; De Wilde, 1971). To the south, *Adenia* species, such as *A. repanda* and *A. gummifera*, are found as far as South Africa, reaching as far south as approximately 30° and 33° latitude, respectively (De Wilde, 1971). In Madagascar, *Adenia* species are found in arid and wet regions, from the lowlands to the central highlands. The genus also thrives in various habitats, ranging from the driest deserts of coastal Namibia to the humid rainforests of Southeast Asia (De Wilde, 1971; Hearn, 2006). In the Flora of Southern Africa, which includes South Africa, Namibia, Botswana, Lesotho, and Swaziland, the genus *Adenia* is represented by ten species and 13 taxa (Crouch et al., 2016). Among these, *Adenia gummifera* is notable as one of the two infraspecific varieties of *A. gummifera*, native to a range from southwest Ethiopia to South Africa and the Seychelles. This species is a tall climber with somewhat woody stems that grow in altitudes of 50 m. The leaves of *A. gummifera* are typically entire or deeply lobed (3-5 lobes), ovate or orbicular in shape, featuring a truncated base (Rotich, 2022).

### 3.2 Ethnobotany and medicinal uses

While many species of *Adenia* are toxic and have been used in traditional practices for poisoning or hunting, some are utilized in conventional medicine across various regions for ailments ranging from common illnesses to more severe conditions (Sarkodie et al., 2013; Sinei, K. A. and Mwangi, 2015; Barua et al., 2020; Maroyi, 2020; Maroyi, 2020; Rotich, 2022; Chibuye et al., 2024). For instance, *A*. *gummifera* is particularly recognized for its traditional medicinal uses throughout tropical Africa, occurring naturally in countries such as South Africa, the Democratic Republic of Congo, Ethiopia, Eswatini, Kenya, Mozambique, Malawi, Seychelles, Somalia, Uganda, Tanzania, Zimbabwe, and Zambia. It typically grows on rocky slopes, termite mounds, and ravines, adapting to various ecological niches (Maroyi, 2020). It is well-known as Snake climber (English) and also called kinyelwet in the Kipsigis community of Kenya; imfulwa in the Zulu community in South Africa; Muhore in the Shona community of Zimbabwe; and Mandali or Ngole in Suaheli or Sukuma communities respectively in Eastern Africa (Luman and Mottram, 2006; Adedapo et al., 2008; Rotich et al., 2021; Jambwa et al., 2022; Rotich, 2022). The infusions of this plant are used as emetics and for treating depression (Adedapo et al., 2008). The roots are part of an herbal concoction called CareVid, serves as an immune booster and health tonic for treating and managing HIV/AIDS opportunistic infections in Kenya (Maroyi, 2020; Rotich, 2022). These roots are also employed as a disinfectant or diuretic and traditional medicine for ailments like respiratory infections, filariasis, Oral candidiasis, sexually transmitted infections, gastrointestinal infections, and infertility (Fullas et al., 1995; Maroyi, 2020; Rotich et al., 2021). In nearby Mozambique, a root infusion is prepared by boiling a six-inch section of the root in three to four pints of hot water (100°C) for consumption by the patient to treat malaria and leprosy, often inducing vomiting and perspiration (Luman and Mottram, 2006). The entire plant can be pounded and dissolved in drinking water to treat lethargic birds, coughing, flu-like symptoms, Newcastle disease, coccidiosis, and blindness (Jambwa et al., 2022). In Tanzania, the juice derived from the leaves of this plant is used to alleviate colic, while crushed leaves are used topically to help heal broken bones (Fullas et al., 1995). However, hepatic toxicity has been associated with the chronic traditional use of *A.* leaves among the Zulu of South Africa (Fullas et al., 1995; Luman and Mottram, 2006). The leaves are also cooked as a vegetable in tropical Africa (Maroyi, 2020). The stems and roots of the plant are sold as traditional remedies in informal herbal medicine markets in Mozambique, Botswana, Gauteng, and KwaZulu-Natal provinces in South Africa (Maroyi, 2020). The stems of *A. gummifera* are combined with other plants to create a mixture for protective charms (Luman and Mottram, 2006).

Another most used plant is *A. cissampeloides*, which is commonly known as snake climber or monkey rope, and locally called Muore in Zimbabwe, Arokeke or Godogbo among the Yoruba in Nigeria (Ishola et al., 2015; Ngarivhume et al., 2015). It thrives in humid areas of West Africa, distributed from Senegal to Somalia, with a notable presence in Ghana and Nigeria (Agbor and Okoi, 2014; Uno et al., 2018; Uzoeto et al., 2018). This plant has a wide range of traditional medicinal applications, including cold or hot infusions of the root or bark for treating malaria and fever (Ngarivhume et al., 2015). It is also widely used as a fishing poison by various tribes from Senegal to Tanzania; in Nigeria, fresh leaves are roasted, pounded, and thrown into rivers, resulting in small fish floating dead within 15 minutes and larger fish within an hour (Neuwinger, 2004; Agbor, R.P. and Okoi, 2014; Ibiam et al., 2015; Uno et al., 2018). The stem is used in Nigeria as fish poison when macerated in water and as emetic for biliousness (Ibiam et al., 2015). In Ghana, it treats malaria, dysentery, rheumatic pain, hypertension, and numbness (Annan et al., 2012), while its leaves are used as a vegetable, and the sap serves as a cosmetic (Ibiam et al., 2015; Ogunlesi et al., 2019). In Ivory Coast, the leaf extract is applied to the breast after childbirth to encourage lactation (Ibiam et al., 2015). Decoctions and infusions of the leaves, stem, or root are employed for gastrointestinal disorders like constipation and diarrhea, as well as rheumatic pain and wound dressing (Nyansah et al., 2016; Ekpo et al., 2017). These decoctions or infusions are also used to address various inflammatory conditions, including edema and rheumatism, as well as to provide pain relief, particularly for headaches and back pain. Moreover, pounded roots are commonly used to dress sores and wounds (Ekpo et al., 2017). The decoction made from the leaves or roots is consumed as a diuretic and to treat malaria and fever (Ekpo et al., 2017). For leprosy, a decoction made from the leaves is applied directly to the sores, while an oral intake of root decoction is combined with a steam bath using the leaves. To treat scabies, ashes from the root or bark are combined with castor oil. A root decoction is consumed to manage cholera and is taken with milk to address anemia in eastern Africa. An extract from the roots and stems is used orally for intestinal worms, and a decoction from leaves is consumed for liver issues. A paste made from leaves is used on fractures and broken bones in Tanzania (Ekpo et al., 2017). Additionally, leaf infusions serve as stimulants for depression and insanity in Zimbabwe, with the inner bark used by the Mano people of Liberia to induce amnesia (Ishola et al., 2015). The plant is noted for its antiplasmodial and antimicrobial properties (Agbor and Okoi, 2014; Nyansah et al., 2016). While it possesses toxic properties, particularly due to the gum resin containing modeccin, *A.* cissampeloides remains economically important for its diverse medicinal uses and fishing practices (Ibiam et al., 2015).

Additionally, *A*. *lobata* is a species widely utilized in traditional medicine across African regions. It extends from Senegal to Angola and Cameroon, and is particularly used in Côte d'Ivoire, Ghana, and eastern Nigeria. It is a liana that is often employed for the treatment of various ailments, including jaundice, malaria, headaches, abdominal pains, otitis, coughs, infantile asthma, rheumatic, respiratory disorders, syphilis, gonorrhea, and even nose cancer (Koffi Marcel et al., 2011; Agoreyo et al., 2012; Bertrand Fowa et al., 2019; Fowa et al., 2019; Garba Koffi et al., 2021). In Ivorian traditional pharmacopeia, *A. lobata* is also used to alleviate anemia in children and pregnant women, relieve gingival inflammation, facilitate labor, and treat malaria fever (N’Go et al., 2021; Florian et al., 2022). In Ghana, it is used to treat diabetes mellitus (Sarkodie et al., 2013), while in some eastern communities of Nigeria, the plant extracts are used for rheumatic pain, headaches, abdominal pain, and cancer (N’Go et al., 2021). Moreover, the plant is recognized for its antihyperglycemic and antioxidant properties (Bertrand Fowa et al., 2019; Fowa et al., 2019).

*A*. *trilobata*, locally referred to as akandaphal in Bangladesh, is distributed in the Chittagong district, as well as in Assam, Pakistan, Myanmar, and the western and eastern Himalayas (Barua et al., 2020). Traditionally, it is used in Bangladesh (South Asia) for various herbal treatments, particularly for diabetes, arthritis, toxicity, and other age-related diseases (Islam et al., 2022). The poultice made from its leaves is applied to alleviate headaches, stomach issues, knee pain, and snake bites (Aziz et al., 2020; Barua et al., 2020).

*A. glauca*, commonly referred to as “Elephant foot,” is a climbing, tuberous, perennial shrub native to the dry bushveld of southern Africa (Immaculate et al., 2022; 2023). This plant is primarily found in rocky areas and has been noted to be poisonous to cattle; however, children in Africa have been observed to consume the fruit without adverse effects (Spencer K. C. and Seigler D. S., 1982). The leaves at the plant’s base are cyanogenic, while the tuber is non-cyanogenic and considered non-poisonous (Spencer K. C. and Seigler D. S., 1982). Traditionally, the powdered form of *A. glauca* has been utilized to treat various medical conditions, including ear infections, swollen legs, and skin diseases. The preparation typically involves grinding the plant material into a powder, which is then applied as a topical treatment (Immaculate et al., 2023). Furthermore, the plant is known to contain galactose-binding lectins and extracts that are useful for protein synthesis and hemagglutinating activity, highlighting its potential as a therapeutic resource (Immaculate et al., 2022).

*A. volkensii* is a perennial shrub found growing wild in several regions of Kenya. The plant is particularly noted for its high toxicity and is traditionally used to kill hyenas, which is reflected in its local Kamba name “kiliambiti,” meaning “eater of hyenas” (Gondwe et al., 1978; Barbieri et al., 1984). Documented cases of poisoning have been reported in Kenya, where individuals became ill after consuming a local gruel that was reportedly contaminated with an extract of *A. volkensii* (Gondwe et al., 1978).

*A. digitata*, commonly known as Modecca, is a tuberous, perennial shrub that thrives in dry regions of South Africa. Both the tuber and fruit are known to be highly toxic to humans, with cases of death reported following accidental, suicidal, or homicidal ingestion (Spencer K. C. and Seigler D. S., 1982).

*A. panduriformis* is a woody climber plant primarily found in Zambia, Mozambique, Tanzania, and Zimbabwe. It could thrive in similar climatic conditions elsewhere in the world.

The plant is a wild vegetable that is commonly consumed as part of the daily diet in various Zambia communities. It is also utilized in traditional medicine to treat and manage a range of diseases, such as degenerative conditions. Traditional healers often prescribe *A. panduriformis* to patients with weakened immune systems, including those living with HIV/AIDS, as it is believed to be an excellent immune booster. Additionally, this leafy vegetable is claimed to have healing properties for several ailments, including diabetes, hypertension, malaria, and diarrhea (Chibuye et al., 2024).

*A. wightiana* is a tuberous, perennial liana characterized by axillary, branched tendrils. It is widely recognized in folk medicine for its medicinal properties, particularly in treating ailments like peptic ulcers. The leaves of this plant are also consumed as a vegetable by the tribes residing in the Anamalai Hills of the Western Ghats (Presena and Pragasam, 2016).

*A. viridiflora* is one of the medicinal plants of the *Adenia* genus used traditionally to treat fever, urinary tract infection, diarrhea, giddiness, and fainting. Its extracts show potential in managing conditions such as obesity, diabetes, hypertension, and Alzheimer’s disease. The edible parts of *A. viridiflora* include the leaves, flowers, young fruits, and shoots which are typically fermented or blanched using washing rice water and served with a spicy sauce as a vegetable accompaniment (Wannasaksri et al., 2021a).

*A. globosa* Engl. is also used traditionally in Kenya, where freshly prepared juice from the tuber is administered to cattle, cows, and goats to facilitate and expedite parturition, particularly for those experiencing difficulties during delivery (Sinei and Mwangi, 1995; Sinei, and Mwangi, 2015). Additionally, an infusion made from this tuber has been shown to induce dose-dependent contractions of the rat uterus, further supporting its traditional use in promoting faster parturition in domestic animals (Gruber and OʼBrien, 2011).

### 3.3 Phytochemistry

*Adenia* species are also known for their rich phytochemical profile. These plants contain a diverse array of secondary metabolites that have been explored for their medicinal properties. Phytochemical studies on different species of *Adenia* such as *A. cissampeloides*, *A. gummifera*, *A. lobate*, *A. panduriformis*, *A. trilobata*, and *A. volkensii* have revealed a variety of compound classes. These include alkaloids, flavonoids, tannins, phenols, steroids, terpenoids, polyphenols, saponins, carbohydrates, proanthocyanins, glycosides, and even potentially toxic proteins like type 2 ribosome-inactivating proteins (RIPs) (Gondwe et al., 1978; Severino et al., 2009; Okem et al., 2014; Bertrand Fowa et al., 2019; Ogunlesi et al., 2019; Barua et al., 2020; Maroyi, 2020; Rotich, 2022; Chibuye et al., 2024). The investigation of some of these species led to the isolation of 27 compounds belonging to different classes. These include six cyanohydrin glycosides, one alkaloid, one ceramide, six flavonoids, three steroids, one triterpene, two fatty acids, one polyacetylene, and six type 2 RIPs. In addition, 17 compounds were found in the essential oils obtained from *A. cissampeloides* leaves. Furthermore, some heavy metals including aluminum, chromium, cadmium, copper, iron, manganese, lead, nickel, mercury, zinc, and tin were also found in an *Adenia* species (*A. gummifera*) (Rotich, 2022).

#### 3.3.1 Cyanohydrin glycosides, alkaloid and ceramide

**FIGURE 2:**
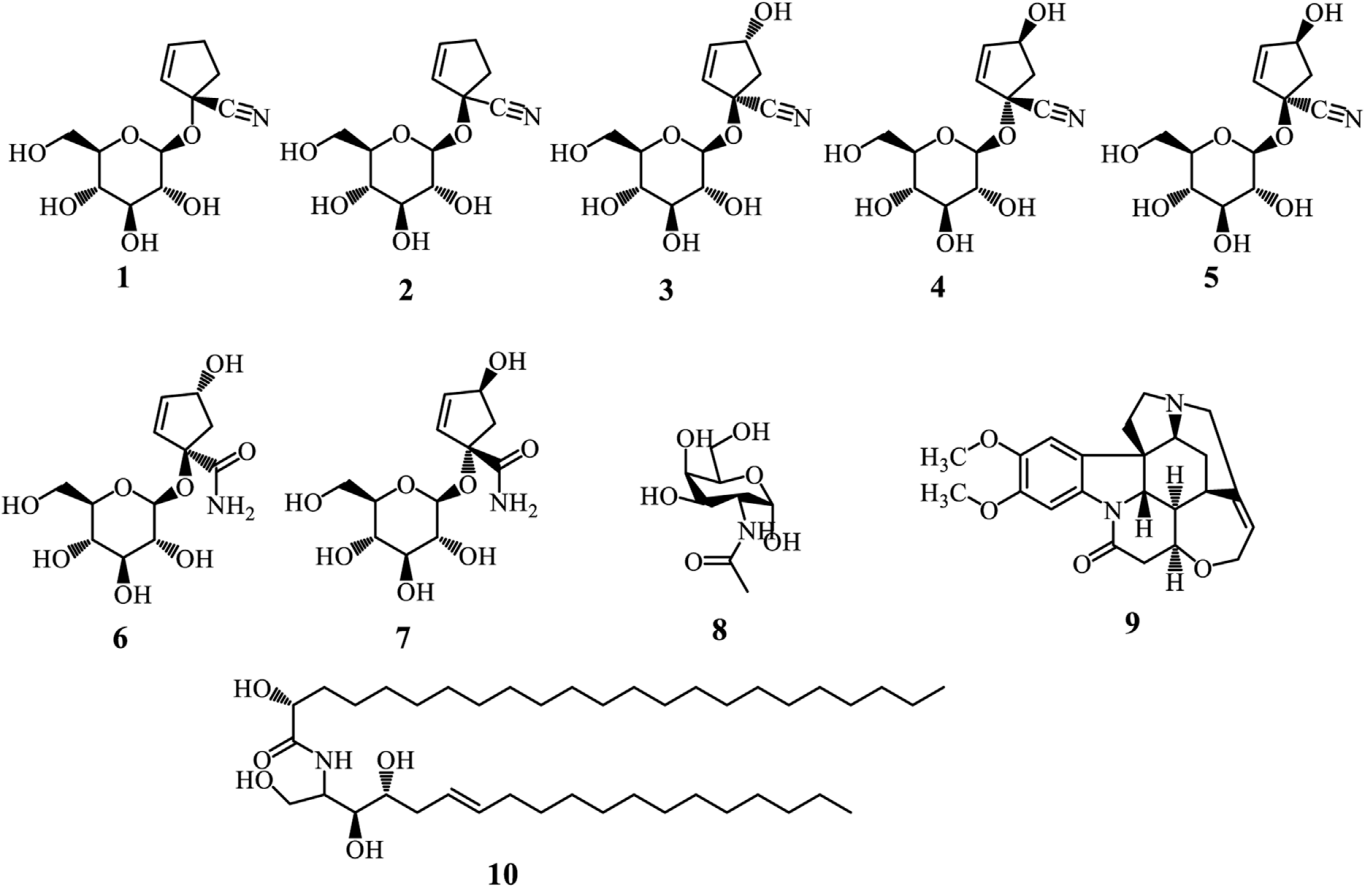
Cyanohydrin glycosides (1-8), Alkaloid (9), and Ceramide (10) from *Adenia* species.

Cyanohydrin glycosides are unique plant compounds defined by a cyanohydrin group attached to a sugar moiety, forming a glycosylated structure. These compounds originate from amino acids and are crucial for plant defense, particularly against herbivores, as they can release toxic hydrogen cyanide when hydrolysed enzymatically (Jørgensen et al., 2011; Gleadow and Møller, 2014). The biosynthesis of cyanohydrin glycosides involves several enzymatic steps, primarily catalysed by cytochrome P450 enzymes. The process typically begins with the conversion of amino acids into oximes, which are then transformed into cyanohydrins through the action of specific enzymes (Jørgensen et al., 2011; Gleadow and Møller, 2014). Alkaloids are a diverse group of naturally occurring organic compounds that primarily contain basic nitrogen atoms. Six cyanohydrin glycosides were isolated from the *Adenia* genus (Figure 2). These include tetraphyllin A (**1**) and deidaclin (**2**) isolated from *A. globosa* (Sinei and Mwangi, 1995), volkenin (**3**) from *A. volkensii* roots (Jaroszewski et al., 1987), tetraphyllin B (**4**) and epi-tetraphyllin B (**5**) from the fresh tubers of *A. digitate*, *A. volkensii*, *and A. glauca* (Gondwe et al., 1978; Spencer K. C. and Seigler D. S., 1982; Jaroszewski et al., 1987). Compound (**4**) was also isolated from the roots of *A. cissampeloides* (Ogunlesi et al., 2019). Importantly, (Jaroszewski et al. (1987) demonstrated compounds (**3**) and (**3**) can be converted into (1R,4R)- (**7**) and (1S,4S)-1-(β-D-glucopyranosyloxy)-4-hydroxy-2-cyclopentene-1-carboxamides (**7**) respectively, through a series of reactions, including alkaline hydrolysis and spontaneous lactonization in methanol (Jaroszewski et al., 1987). Another cyanohydrin glycoside is N-Acetyl galactosamine (**8**) isolated from *A. hondala* (Sharma et al., 2018). However, brucine (**9**), isolated from the leaves of *A. lobata*, is the only alkaloid reported from the genus (Agoreyo et al., 2012). At the same time, adeniamide (**10**) was the only ceramide isolated from the stem bark of *A. lobata* (Garba Koffi et al., 2021).

#### 3.3.2 Flavonoids

**FIGURE 3:**
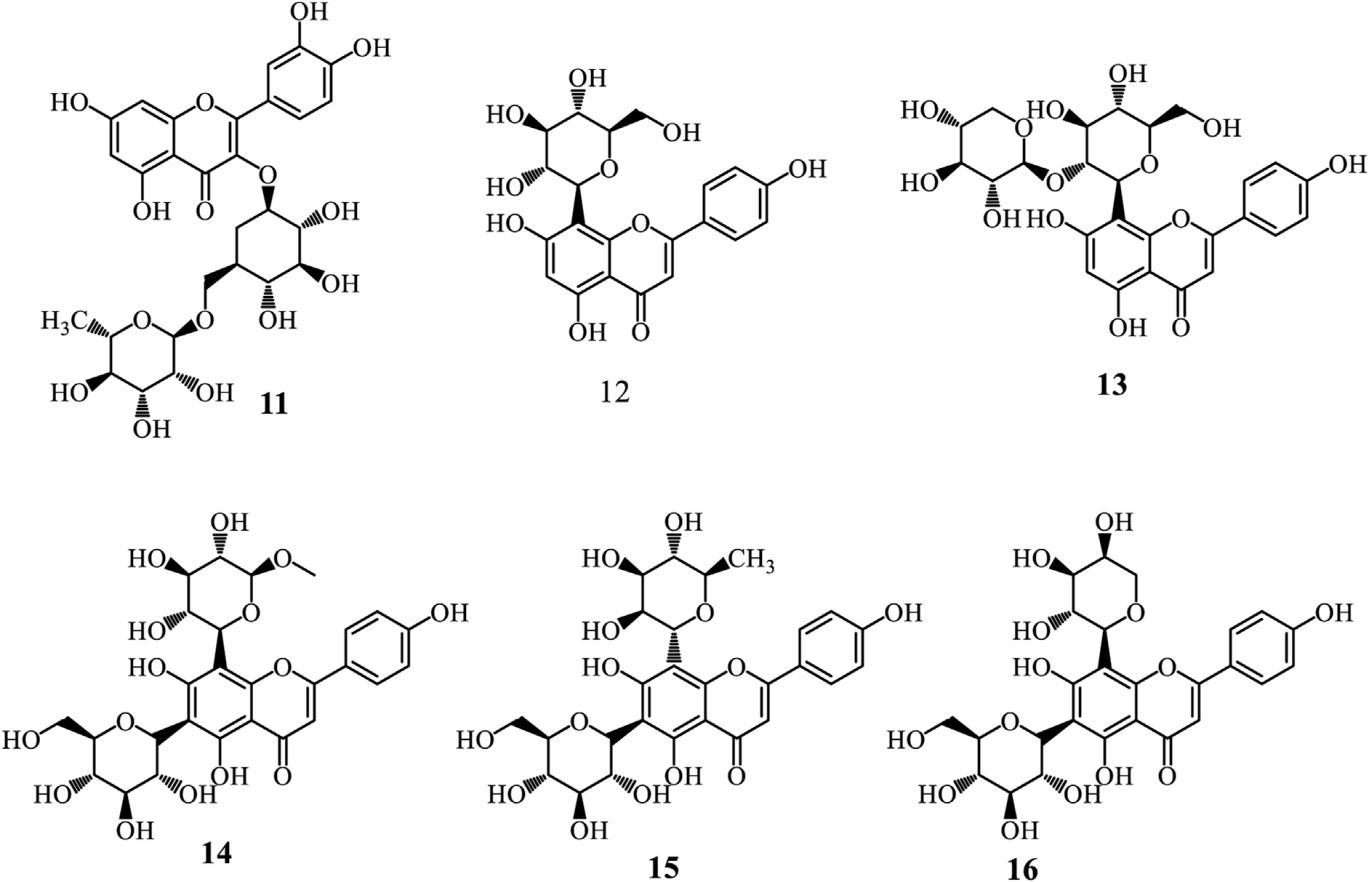
Glycosyl flavonoids from *Adenia* species.

Flavonoids represent a diverse group of natural polyphenolic compounds with a basic structure of 2-phenyl-benzopyran, consisting of two aromatic rings (A and B) linked by a heterocyclic pyran ring (C) containing an oxygen atom (Falcone Ferreyra et al., 2012; Yang et al., 2018; Liga et al., 2023). Glycosyl flavonoids are bonded to sugar molecules, enhancing their solubility and bioavailability (Liga et al., 2023). The flavonoid biosynthesis occurs through the phenylpropanoid pathway, starting with phenylalanine. Key enzymatic steps include the transformation of phenylalanine into trans-cinnamic acid by phenylalanine ammonia-lyase (PAL), followed by its transformation into 4-coumaric acid and then into 4-coumaroyl-CoA. Chalcone synthase catalyses the condensation of 4-coumaroyl-CoA with malonyl-CoA to produce chalcone, which can be further modified into various flavonoid subclasses (Falcone Ferreyra et al., 2012; Yang et al., 2018; Liga et al., 2023). Glycosylation occurs via glycosyltransferases, attaching sugar moieties to the flavonoid backbone, resulting in glycosyl flavonoids with distinct biological activities (Falcone Ferreyra et al., 2012). Only six glycosyl flavonoids have been isolated from the genus *Adenia* (Figure 3). These include Rutin (**11**) isolated from *A. glauca* roots (Immaculate et al., 2022), vitexin (**12**), 2″-xylosylvitexin (**13**), and a mixture of vicenin-2 (**14**), violanthin (**15**), and schaftoside (**16**) isolated from the leaves of *A. mannii* (Ulubelen and Oksuz, 1983).

#### 3.3.3 Steroids and triterpenes

**FIGURE 4:**
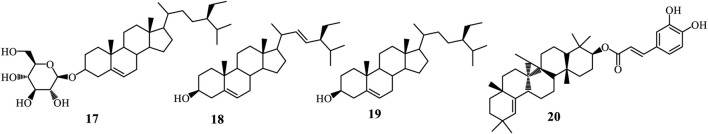
Steroids and Triterpenes from *Adenia* species.

Steroids are organic compounds defined by a core structure of four fused carbon rings, known as the steroid nucleus. Their biosynthesis primarily derives from cholesterol through the mevalonate pathway, which begins with acetyl-CoA converting to mevalonic acid via HMG-CoA reductase. Mevalonic acid is then transformed into isoprenoid units and assembled into squalene, which undergoes cyclization to form lanosterol. This precursor is further modified to produce various steroid hormones (Harrison, 1985). Triterpenes, composed of six isoprene units and resulting in a 30-carbon skeleton, are synthesized via the same mevalonate pathway. The process starts with the formation of isopentenyl pyrophosphate (IPP) from acetyl-CoA, followed by the condensation of IPP units into squalene. Specific enzymes, such as oxidosqualene cyclases, then catalyze the cyclization of squalene, yielding diverse triterpenoid structures (Harrison, 1985; Lu et al., 2024). These compound classes are extensively found in plants and are recognised for their various biological activities. From *Adenia* species, three steroids have been isolated (Figure 4). These include *β*-sitosterol-3-*O*-*β*-D-glucopyranoside (**17**) and a mixture of stigmasterol (**18**) and *β*-sitosterol (**19**) isolated from *A. lobata* stem bark. Whereas, only one triterpene, germanicol caffeoyl ester (**20**), has been isolated from *A. lobata* stem bark (Garba Koffi et al., 2021).

#### 3.3.4 Fatty acid and polyacetylene

**FIGURE 5:**
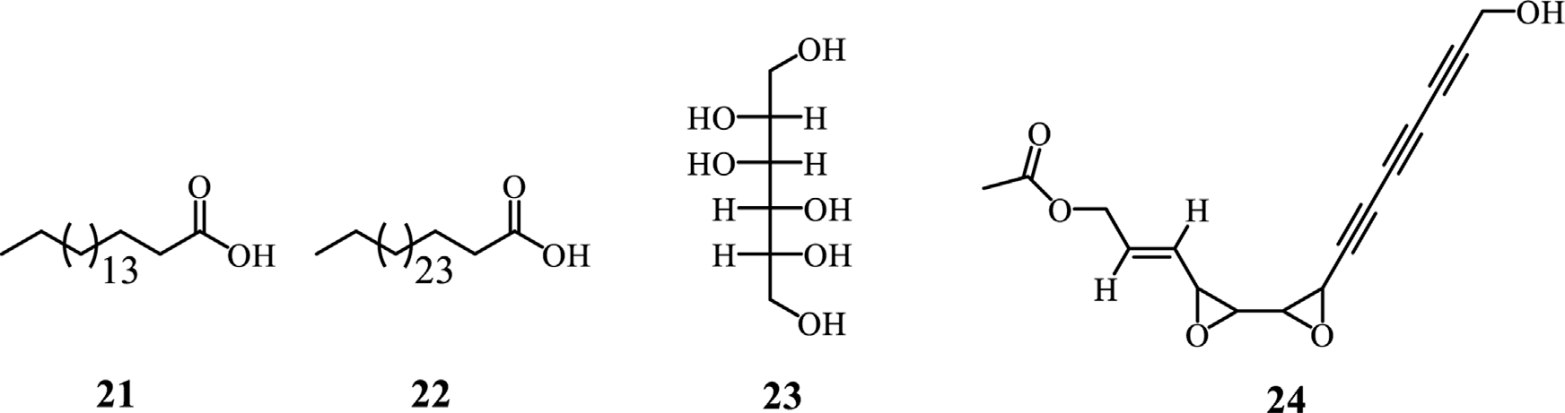
Fatty Acid and polyacetylene from *Adenia* species.

Fatty acids are long-chain carboxylic acids, either saturated or unsaturated, crucial for energy storage, cellular structure, and signaling in organisms. Their synthesis mainly occurs via the fatty acid synthase (FAS) complex, beginning with acetyl-CoA, which is converted to malonyl-CoA and then condensed into a 3-ketoacyl-ACP intermediate. This intermediate undergoes reduction and dehydration, producing saturated fatty acids, with elongation processes resulting in common fatty acids like palmitic and stearic acid (Minto and Blacklock, 2008; Scott et al., 2022; Salomon, 2024). Polyacetylenes, known for their carbon-carbon triple bonds, are derived from fatty acid precursors such as crepenynic and stearolic acids. Their biosynthesis includes desaturation of fatty acids and the formation of acetylenic bonds through oxidative dehydrogenation or decarboxylative enol elimination, leading to diverse polyacetylene derivatives (Minto and Blacklock, 2008; Scott et al., 2022; Salomon, 2024). From *Adenia* species only two fatty acids are reported, including octacosanoic acid (**21**) and D-mannitol (**22**) isolated from *A. lobata* stem bark (Figure 5) (Garba Koffi et al., 2021). Whereas gummiferol (**23**) is the only polyacetylene isolated from *A. gummifera* leaves (Fullas et al., 1995; Luman, F.M., and Mottram, 2006).

#### 3.3.5 Essential oils

**FIGURE 6:**
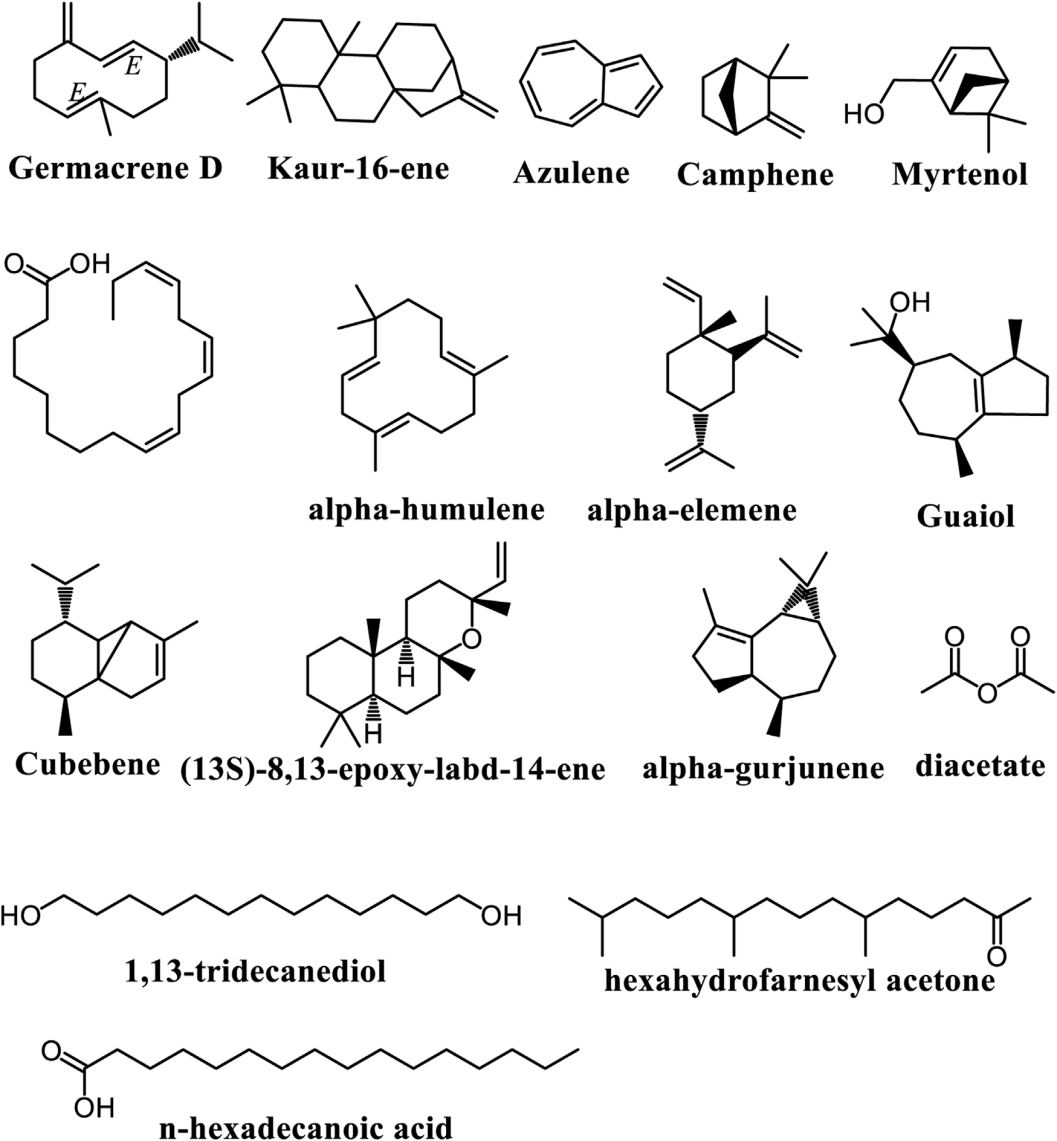
Main volatile chemical constituents found in *Adenia* species.

Essential oils (EOs) are concentrated, hydrophobic liquids derived from plants, known for their strong aromas, and composed mainly of monoterpenes and sesquiterpenes. Typically, EOs contain 20 to 60 components, with two or three major constituents making up 20%–70% of the mixture (Rashidinejad and Jafari, 2020; Santos et al., 2022). Their chemical composition can vary significantly due to factors such as season, climate, water stress, geographic location, soil conditions, and other abiotic influences. In 2019, Ogunlesi and collaborators analysed the EOs from *A. cissampeloides*dried leaves. They found a total of 17 constituents from five essential derived from the species. These include phytol, germacrene D, n-hexadecanoic acid, α-linolenic acid, 1,13-tridecanediol, diacetate, hexahydrofarnesyl acetone, kaur-16-ene, (13S)-8,13-epoxy-labd-14-ene, guaiol, α-elemene, α-gurjunene, α-humulene, myrtenol, camphene, azulene, and cubebene (Figure 6). The major compound in the EO samples was phytol, with other notable constituents including germacrene D, diacetate, and 1,13-tridecanediol which were predominant in the first and second hours of collection, respectively (Ogunlesi et al., 2019).

#### 3.3.6 Type 2 ribosome-inactivating proteins (RIPs)

RIPs are a class of enzymes primarily derived from plants that can inhibit protein synthesis by targeting eukaryotic ribosomes. They are categorized into three main types: type I RIPs, which are single-chain proteins; type II RIPs, which consist of an A-chain and a B-chain; and type III RIPs, which are less common and have additional domains (Stirpe et al., 2007; Bortolotti et al., 2021; 2024; Maiello et al., 2021). Type 2 RIPs, mostly isolated from the *Adenia* genus, are responsible for binding to cell surface receptors and then highly cytotoxic, making them of interest for both therapeutic and research applications. These proteins are purified by using Affinity Chromatography and Gel Filtration techniques and characterized by using SDS/PAGE and ESI-MS techniques (Barbieri et al., 1980; Chambery et al., 2004; Severino et al., 2009; Tosi et al., 2009; Mercatelli et al., 2020; Bortolotti et al., 2021; Bortolotti et al., 2024). Type 2 RIPs from *Adenia* species include modeccin, isolated from *A. digitata* and *A. volkensii* roots. This toxin is a protein with a molecular weight of 57,000, which can dissociated into two subunits weighing 25,000 and 32,000 (Gasperi-campani et al., 1978; Barbieri et al., 1980; Bortolotti et al., 2021; Bortolotti et al., 2024). Another type 2 RIP from *Adenia* species, is volkensin, also isolated from the roots of *A. volkensii*, with a molecular weight of 60,000. The A-chain has a molecular weight of 29,000, while the B-chain displays double molecular weights of 37,400 and 36,000 (Chambery et al., 2004; Severino et al., 2009). Volkensin has additionally been extracted from *A. digitate* roots (Bortolotti et al., 2024) and from the caudex of *A. kirkii* (Bortolotti et al., 2021). Stenodactylin is isolated from the caudex of *A. stenodactyla*, consisting of two chains connected by a disulfide bond. The A-chain acts on 28S rRNA, while the B-chain binds to glycan structures. Its molecular weight is 53,800, reduced into two bands of 24,800 and 30,000 for the A and B chains respectively (Stirpe et al., 2007; Tosi et al., 2009; Mercatelli et al., 2020; Bortolotti et al., 2024). This protein was characterized through crystallization and X-ray diffraction, resulting in crystals that diffract at high resolution, reaching up to 2.15 Å (Tosi et al., 2009). Lanceolin, isolated from the caudex of *A. lanceolata*, has a molecular weight of 63,000, with A and B chains ranging from 27,000 to 33,400 (Stirpe et al., 2007; Bortolotti et al., 2021; 2024). Kirkiin, derived from the caudex of *A. kirkii*, has an approximate molecular weight of 58.5 kDa, which reduces to bands of 27.1 kDa and 35.3 kDa (Bortolotti et al., 2021; 2024). Lastly, heterophyllin from the caudex of *A. heterophylla* corresponds to 60 kDa under non-reducing conditions and reduces to bands of about 25 kDa and 33 kDa (Bortolotti et al., 2024).

### 3.4 Pharmacological properties of *Adenia*

The pharmacological properties of *Adenia* species highlight their potential for therapeutic applications, including antimicrobial, antioxidant, anti-inflammatory, analgesic, anticholinesterase, neuropharmacological, antidepressant, antihyperglycaemic, anti-anemic, anticoagulant, antithrombotic, thrombolytic, and anesthetic, uterine contraction, and cytotoxicity activities (Table 2).

**TABLE 2:** Pharmacological Importance of the genus *Adenia*.

Species	Pharmacological activity	Part uses	Tested extract	Model	Control	Dosage range	Results/effects	References
*A. gummifera* var. gummifera	Antibacterial	Leaves, stem bark	Aqueous	*In vitro*	-	5.0 mg/mL	Active against *Enterobacter cloacae*, *Escherichia coli*, *Pseudomonas aeruginosa*, *Klebsiella pneumoniae*, and *Serratia marcescens*	Rotich (2022)
	Antifungal	Root bark	Aqueous/ethanol	*In vitro* (ZOI/MIC)	-	-	Active against *Candida albicans* and *Rhodotorula spp* (ZOI = 10–37 mm; MIC = 61.0–72 mg/mL)	Maroyi, 2020; Rotich, 2022
	Antiplasmodial	Roots, leaves	Petroleum ether/ethyl acetate (1:1)	*In vitro* (IC_50_)	Chloroquine	50 mg/mL	Active against PoW strain (IC_50_ = 50 μg/mL) and none active against chloroquine-resistant clone Dd2 strain	Kraft et al. (2003)
	Antioxydant	Stem	Methanol	*In vitro* ABTS analysis	BHT	0.08 mg/mL	Showed a total antioxidant power of 159.12 mm Fe(II)/g, with 94.2% inhibition	Adedapo et al. (2008)
	Anticholinesterase	Root	Ethyl acetate	*In vitro* (IC_50_) Ellman’s colorimetric method	Galanthamine	Dose-dependent	Showed inhibition with IC_50_ = 0.02 mg/mL	Adewusi and Steenkamp (2011)
	Anesthetic	Stem	Steam distillates/aqueous	*In vitro* diffusion assays	Isoflurane	Dose-dependent	Showed strong effects on *Apis mellifera*, with the aqueous extract peaking at 6.0% concentration	Ngarivhume et al. (2008)
	Cytotoxicity	Leaves	20% MeOH/CHCl_3_/H_2_O	*In vitro* KB human cell line	-	-	ED_50_ = 1.1 μg/mL	Fullas et al. (1995)
*A. cissampeloides* (Planch. ex Hook.) Harms	Antibacterial	Leaves	Petroleum ether/methanol	*In vitro* Agar diffusion (ZOI)	ciprofloxacin (5 µg)	Dose-dependent	The petroleum extract active against *Aeromonas veronii* (ZOI = 8.2 mm), while the methanol extract was active against *Raoutella ornithinolytica* (ZOI = 7 mm) and *Aeromonas hydrophila* (ZOI = 5 mm)	Uzoeto et al. (2018)
	Antiplasmodial	Stem	Ethanol	*In vitro* *Plasmodium falciparum* (IC_50_)	Chloroquine	1.562 μg/mL	Demonstrated maximum growth inhibition of 73.95%, with an IC_50_ value of 8.521 μg/mL	Annan et al. (2012)
	Antidepressant	Leaves	Hydroethanol	*In vivo* (Mice) FST, TST, HBT behavioral tests	Fluoxetine, sulpiride, yohimbine	1000 and 200 mg/kg	Significantly reduced immobility in FST, increased swimming activity by 75.20% and climbing by 190.00%. In TST, the extract decreased immobility time by 35.60% and 35.27%	Ishola et al. (2015)
	Anxiolytic	Leaves	Hydroethanol	*In vivo* (Mice) HBT behavioral tests	Cyproheptadine	50–200 mg/kg	Increased head dips by 41.67% and open-arm entries in the elevated plus maze (p < 0.001)	Ishola et al. (2015)
	Anticoagulant	Aerial parts mix with the root bark of *Pseudocedrela kotschyi*	Aqueous	*In vivo* (New Zealand White rabbits) aPTT and PT models	Rivaroxaban, aspirin, heparin, dalteparin	0.5–2.0 g/L	Not significantly affect PT (P > 0.05) but significantly increased aPTT (P ≤ 0.05–0.0001).	Nyansah et al. (2016)
				*In vivo* (Sprague-Dawley rats) CT and BT models	Rivaroxaban, aspirin, heparin, dalteparin	250–2000 mg/kg	Significantly prolonged BT and CT (P ≤ 0.05–0.0001).	Nyansah et al. (2016)
	Antithrombotic	Aerial parts mix with the root bark of *Pseudocedrela kotschyi*	Aqueous	*In vivo* (Sprague-Dawley rats) carrageenan-induced thrombosis model	Heparin	500 and 2000 mg/kg	Significantly reduced tail infarction and inflammation (P ≤ 0.01–0.001). Showed a notable decrease in microthrombi count in the lungs and liver (P ≤ 0.0001)	Nyansah et al. (2021)
	Toxicity	Stem bark, leaves	Aqueous/methanol/ethanol	*In vivo* (Catfish)	-	Dose-dependent	Stem bark aqueous (LC_50_ = 5.0 g/L (24 h), 2.5 g/L (48 and 72 h)), methanol (LC_50_ = 50 mg/L in 72 h); leaf 70% ethanol (LC50 = 42.38 mg/L in 96 h)	Agbor and Okoi, 2014; Ibiam et al., 2015; Ekpo et al., 2017; Uno et al., 2018
*A. lobata* (Jacq.) Engl.	Anti-*β*-lactamase	fruits, seeds, leaves, stems, bark, roots, twigs	methylene chloride/methanol (1:1)	*In vitro* TEM-1 β-lactamase enzyme	phosphate buffer	Dose-dependent	90% inhibition	Gangoué-Piéboji et al. (2007)
	Antileishmanial	Stem bark, leaves	CH_2_Cl_2_–MeOH (1:1), ethyl Acetate, methylene chloride, methanol	*In vitro* *Leishmania donovani* promastigotes	Amphotericin B	Dose-dependent	Stem bark CH_2_Cl_2_–MeOH (1:1) active (IC_50_ = 21.17 μg/mL) and its ethyl acetate fraction (IC_50_ = 34.42 μg/mL), leaf methylene chloride active (EC_50_ = 50 μg/mL), while the leaf methanol ineffective (EC_50_ > 100 μg/mL)	Garba Koffi et al. (2021)
		Compound (**20**)	Ethyl acetate	*In vitro* *Leishmania donovani* promastigotes	Amphotericin B	50 μg/mL	Active with IC_50_ of 6.03 μg/mL	Garba Koffi et al. (2021)
	Antioxydant	Leaves	Hydroethanol	*In vivo* (albino rats) Blood culture method	ciproflaxacin	Dose-dependent	*S*ignificantly reduced malondialdehyde levels (p < 0.05) and increased (p < 0.05) superoxide dismutase, glutathione, catalase, and nitric oxide levels	Bertrand Fowa et al. (2019)
		Leaves	70% ethanol, petroleum ether	*In vitro* Total phenolic contents and DPPH tests	N-propyl gallate	Dose-dependent	Total phenolic contents of 0.623 mg/g and 0.513 mg/g; and DPPH IC_50_ values of 0.5210 mg/mL and 2.106 mg/mL for ethanol and petrleum respectively	Sarkodie et al. (2013)
		Cordate leaves	Ethyl acetate	*In vitro* DPPH method	-	Dose-dependent	High activity at 63.87%	Agoreyo et al. (2012)
	Antisalmonellal	Leaves	70% ethanol	*In vitro* liquid microdilution method	Ciprofloxacin	Dose-dependent	Active with MICs values ranging from 8 to 64 μg/mL on *Salmonella Typhi*, *S. Paratyphi* A*, S. Paratyphi* B*, S Typhimurium* and *S Typhi* ATCC 6539	Bertrand Fowa et al. (2019)
	Anti-anaemic	Leaves	Aqueous and 70% hydroethanolic	*In vivo* (anemic rats) hematological parameters, hemoglobin and hematocrit levels	Sodium chloride	250 mg/kg	Aqueous and 70% hydroethanolic extracts significantly improved hematological parameters, with recovery rates of 79.66% and 78.33% respectively; the hemoglobin levels increased significantly to 82.51% and 81.97% respectively; while the hematocrit levels rose to 96.42% and 93.95% respectively	Florian et al. (2022)
	Antihyperglycemic	Stem bark	70% ethanol, petroleum ether	*In vivo* (rats)	Glibenclamide	Dose-dependent	Significant active (p < 0.001); the ethanol extract reduced blood glucose levels by 71.4%, 78.2%, and 82.8% at doses of 150, 300, and 600 mg/kg, while the petroleum ether extract reduced levels by 53.1%, 71.8%, and 77.4% at the same doses	Sarkodie et al. (2013)
*A. trilobata* (Roxb.) Engl.	Anti-diarrhea	Leaves, stem	Methanol, n-hexane	*In vivo* (Mousse)	loperamide	200 and 400 mg/kg	Methanol significantly reduced defecation (P < 0.0001) and diarrhea (P < 0.001) while the n-hexane at 400 mg/kg reduced intestinal motility to 48.79 (P < 0.0001)	Barua et al. (2020)
	Antioxydant	Leaves	Methanol, n-hexane	*In vitro* DPPH scavenging	-	500 μg/mL	The extracts are active activity (P < 0.05), the n-hexane fraction achieving 89.19% scavenging activity with IC_50_ of 139.65 μg/mL	Barua et al. (2020)
	Analgesic	Leaves	Methanol	*In vivo* Formalin-induced and acetic acid-induced writhing tests.	diclofenac Na	400 mg/kg	Significantly reduced writhing with 57.85% inhibition in the acetic acid assay, and with 60.83% and 61.65%; in formalin tests in early and late phases respectively	Barua et al. (2020)
	Anxiolytic	Leaves	Methanol	*In vivo* (Swiss albino mice) elevated plus maze (EPM) and hole-board test (HBT)	diazepam	400 mg/kg	Active with maximum open arm entries of 30.06% and 33.85% respectively	Aziz et al. (2020)
	Thrombolytic	Stem	Methanol	*In vitro*	streptokinase	10 mg/mL	Active by achieving a clot lysis percentage of 25.58% (P < 0.0001)	Barua et al. (2020)
	Cytotoxicity	Stem, leaf	Methanol, n-hexane	*In vivo* brine shrimp (*Artemia salina*) bioassay	vincristine sulfate	2.16 μg/mL	Stem methanol (LC_50_ = 328 μg/mL) and leaf n-hexane (LC_50_ = 616.85 μg/mL)	Barua et al. (2020)
*A. glauca* Schinz	Antioxydant	Compound (**11**)	-	*In vitro* DPPH assay	Ascorbic acid	20–100 μg/mL	Active with percentage inhibitions of 22%–82% and IC_50_ value of 51.56 μg/mL	Immaculate et al. (2022), Immaculate et al. 2023
*A. volkensii* Harms	Cytotoxicity	Roots	volkensin, a RIP	*In vivo* (Mice) Rabbit reticulocyte lysates and HeLa cells	-	0.1–10 µg/100 g bw	Inhibits protein synthesis in cells with LD_50_ = 1.38 μg/kg bw	Barbieri et al. (1984)
*A. digitata* (Harv.) Engl.	Toxicity	Fruits, tuber	Modeccin a RIP	*In vivo* (rats) Rabbit reticulocyte lysates	-	1 µg/100 g bw	High toxic with LD_50_ = 0.31 μg/mL all rats	Barbieri et al. (1980)
*A. panduriformis* Engl.	Antioxydant	Leaves	methanol	*In vitro* DPPH free radical quenching	Ascorbic acid	20–120 μg/mL	Active with an IC_50_ value of 53.48 μg/mL	Chibuye et al. (2024)
*A. viridiflora* Craib	Antioxydant	Young shoots, old leaves		*In vitro* DPPH scavenging, FRAP-reducing, ORAC	Trolox	Dose-dependent	Active with DPPH of 0.78–1.44 µmol, FRAP-reducing ranging from 14.36 to 42.90 µmol, and ORAC varied from 592.08 to 1438.85 μmol TE/g DW	Wannasaksri et al. (2021a); Wannasaksri et al. (2021b)
*A. lanceolata* Engl.	Toxicity	-	Lanceolin a RIP	*In vivo* (mice) Apoptosis in cells	-	10^−10^ M to 10^−12^ M	Highly toxic with LD_50_ = 8.16 mg/kg	Stirpe et al. (2007)
*A. stenodactyla* var. kondensis Harms	Toxicity	-	Stenodactylin a RIP	*In vivo* (mice) Apoptosis in cells	-	10^−10^ M to 10^−12^ M	Highly toxic with LD_50_ = 1.21 μg/kg	Stirpe et al., 2007; Tosi et al., 2009
*A. heterophylla* (Blume) Koord.	Cytotoxicity	-	heterophyllin (RIP)	*In vitro* NB100, T24, MCF7 human-derived cell lines	saporin and ricin	Nanomolar concentrations	Induces apoptosis without necrosis in NB100, T24 cells (EC_50_ = 10^−11^ M). Complete cell killing occurs at 10^−9^ M for NB100 and T24 cells and 10^−10^ M for MCF7 cells	Bortolotti et al. (2024)
*A. hondala* (Gaertn.) de Wilde	Cytotoxicity	-	Lectin	*In vitro* rabbit erythrocytes and human hepatocellular carcinoma HepG2 cells,	-	Dose-dependent	Exhibits non-specific agglutination of cells, with an IC_50_ of 4.8 μg/mL at 72 h. It inhibited HepG2 cell growth by 76% at 72 h	Sharma et al. (2018)

#### 3.4.1 Antimicrobial activitiesaaaaa

*Adenia* species exhibit notable antimicrobial properties, supported by multiple studies. *A. gummifera* demonstrates broad-spectrum antibacterial activity against Gram-negative pathogens like *Escherichia coli* and *Klebsiella pneumoniae* using acetone extracts (5.0 mg/mL) (Rotich, 2022), while its ethanol and aqueous root bark extracts show strong antifungal effects against *Candida albicans* (37.0 mm inhibition zones) (Maroyi, 2020; Rotich, 2022). These activities are linked to flavonoids, alkaloids, and ribosome-inactivating proteins (RIPs) that disrupt microbial membranes, DNA synthesis, and protein synthesis (Tosi et al., 2009; Battelli et al., 2010). Although *A. gummifera* exhibits moderate antiplasmodial activity against chloroquine-sensitive *Plasmodium falciparum* (IC_50_ = 50 μg/mL), it lacks efficacy against resistant strains (Kraft et al., 2003). In contrast, *A. lobata*’s hydroethanolic leaf extract clears *Salmonella typhi* infections in rats within 8–12 days (Fowa et al., 2019) and inhibits TEM-1 β-lactamase by 90%, suggesting potential to counter antibiotic resistance (Gangoué-Piéboji et al., 2007). Its ethyl acetate stem bark fraction and a purified compound (**20**) (IC_50_ = 6.03 μg/mL) show potent antileishmanial activity against *Leishmania donovani*, outperforming less effective methanol extracts (Garba Koffi et al., 2021; Okpekon et al., 2004). Structure-activity relationships highlight hydroxyl/acetyl groups and nitrogen-containing alkaloids as key drivers of efficacy (Gangoué-Piéboji et al., 2007; Garba Koffi et al., 2021). In addition, *A. trilobata* stands out for its gastrointestinal benefits: methanol leaf extracts (200–400 mg/kg) significantly reduced diarrhea episodes and intestinal motility in mice, with the 400 mg/kg dose outperforming the standard drug loperamide in motility inhibition—a promising finding for natural antidiarrheal agents (Barua et al., 2020). Meanwhile, *A. cissampeloides* shows dual antimicrobial potential. Its petroleum ether extract effectively combats fish pathogens like *Aeromonas veronii* (8.2 mm inhibition zones), while methanol extracts target *Raoutella ornithinolytica* (7 mm zones), offering potential solutions for aquaculture-related bacterial infections (Uzoeto et al., 2018). Notably, the same species exhibits moderate antiplasmodial activity against drug-resistant *P. falciparum*, achieving 73.95% parasite growth inhibition at low concentrations (1.562 μg/mL), though its IC_50_ (8.521 μg/mL) remains far higher than artesunate’s 0.03 μg/mL benchmark (Annan et al., 2012). Together, these findings position *Adenia* as a multi-target medicinal resource—from gut health to malaria management—with efficacy often rivalling synthetic drugs, though potency gaps for specific applications warrant further research into compound optimization. They underscore *Adenia*’s potential as a source of novel antimicrobial agents, particularly for drug-resistant infections.

#### 3.4.2 Antioxidant, anti-inflammatory, and nutritional properties

*Adenia* species offer a compelling array of antioxidant benefits, as evidenced by extensive research. *A. lobata* stands out, with studies detailing its ability to modulate oxidative stress *in vivo*. For example, a hydroethanolic leaf extract administered to *S. typhi*-infected rats significantly reduced malondialdehyde levels while boosting crucial antioxidant enzymes like superoxide dismutase, glutathione, catalase, and nitric oxide (Fowa et al., 2019). *In vitro* analyses of *A. lobata* stem extracts revealed that ethanol extracts outperform petroleum ether in multiple antioxidant assays, displaying a total phenolic content of 0.623 mg/g, a total antioxidant capacity of 0.687 mg/g, and a reducing power of 0.645 mg/g (Sarkodie et al., 2013). The ethanol extract also exhibited lower IC_50_ values in DPPH scavenging (0.5210 mg/mL) and lipid autoxidation inhibition (0.5985 mg/mL) assays compared to the petroleum ether extract (Sarkodie et al., 2013). Furthermore, an ethyl acetate fraction from the cordate leaf variety of *A. lobata* showed exceptional antioxidant activity (63.87%), suggesting its potential in cancer therapy (Agoreyo et al., 2012). *A. lobata*’s antioxidant potential extends to neuroprotection, reducing pain and anxiety in mice, effects linked to decreased levels of nitric oxide and malondialdehyde and increased levels of non-protein thiols (N’Go et al., 2021). Other *Adenia* species also demonstrate impressive antioxidant capabilities. *A. gummifera* stem methanol extracts exhibited a reducing ability of 159.12 mm Fe (II)/g and acted as potent ABTS radical scavengers, with activity comparable to the synthetic antioxidant BHT (94.2% inhibition for *A. gummifera* vs. 96.8% for BHT at 0.08 mg/mL) (Adedapo et al., 2008). *A. trilobata* n-hexane leaf fractions displayed the highest scavenging activity (89.19% at 500 μg/mL, IC_50_ = 139.65 μg/mL) (Barua et al., 2020), while *A. panduriformis* leaf methanol extracts even surpassed ascorbic acid in DPPH free radical quenching (IC_50_ = 53.48 μg/mL vs. 74.47 μg/mL) (Chibuye et al., 2024). In *A. viridiflora*, older leaves exhibited higher DPPH scavenging (0.96–1.44 μmol TE/100 g DW) and FRAP activities (27.08–42.90 μmol TE/g DW) compared to younger shoots (Wannasaksri et al., 2021b), suggesting an age-related accumulation of protective compounds. Finally, a compound isolated from *A. glauca* demonstrated notable free radical scavenging, with an IC_50_ value of 51.56 μg/mL in the DPPH assay (Immaculate et al., 2022). These antioxidant activities are primarily attributed to flavonoids and phenolic compounds, which neutralize free radicals and boost antioxidant enzymes like SOD and glutathione peroxidase (Sarkodie et al., 2013). Variations in activity among extracts from different species such as *A. trilobata*, *A. gummifera*, *A. panduriformis*, and *A. viridiflora* are linked to the specific antioxidants extracted by different solvents (n-hexane vs. methanol) and the accumulation of compounds like flavonoids and tannins as leaves mature (Adedapo et al., 2008; Barua et al., 2020; Chibuye et al., 2024; Wannasaksri et al., 2021b). These antioxidant properties contribute to antinociceptive and anti-anxiety effects by mitigating oxidative stress and inflammation in neuronal tissues, as demonstrated in studies with *A. lobata* (N’Go et al., 2021).These collective findings underscore the significant potential of *Adenia* species as a source of natural antioxidants for various therapeutic applications, offering a promising avenue for further investigation and development.

Furthermore, the nutritional compositions of old leaves and young shoots from *A. viridiflora* were studied using samples collected from four regions of Thailand during various periods (March-April, May-June, and July-August). The young shoots provided 371.45–387.69 kcal of energy and contained 18.15–21.94 g of protein, while old leaves offered 373.22–386.97 kcal and 16.53–22.68 g of protein, generally showing higher protein levels in young shoots, particularly during May-June. In terms of fat content, young shoots ranged from 0.62 to 4.10 g, compared to 1.66–3.28 g in old leaves. Both parts had similar carbohydrate contents (67.21–74.57 g in young shoots and 67.12–73.53 g in old leaves), but young shoots contained significantly more total dietary fiber (32.89–65.26 g) than old leaves (28.37–51.46 g), especially in samples from July-August. Young shoots had total sugar levels of 8.45–16.16 g, while old leaves contained 11.83–18.22 g, with old leaves generally exhibiting higher sugar content. Vitamin C levels were notable, with young shoots ranging from 573.93 to 1696.46 mg and old leaves from 862.64 to 2240.65 mg, the latter providing 1.1–1.9 times more. Lutein was the predominant carotenoid in both parts, with old leaves having significantly higher levels of both β-carotene (1.7–3.6 times) and lutein (1.4–2.6 times). In terms of minerals, potassium was most abundant, with young shoots containing 1740.09–2549.33 mg and old leaves 1357.16–2144.11 mg, while old leaves showed slightly higher iron content (4.90–8.57 mg versus 4.27–7.08 mg in young shoots). Overall, young shoots offered higher energy, protein, and fat, while old leaves provided greater antioxidant activities, phenolic content, and specific vitamins and minerals, highlighting the unique nutritional benefits of both plant parts (Wannasaksri et al., 2021b). *A. viridiflora* offers nutritional benefits, with young shoots rich in protein and fiber and old leaves packed with vitamin C and carotenoids like lutein and β-carotene for antioxidant protection. These nutritional differences arise from the plant’s growth stages, with young shoots focusing on building new tissues and older leaves accumulating protective compounds against environmental stressors (Wannasaksri et al., 2021b). The presence of these vitamins, carotenoids, and essential minerals contributes to overall human health by supporting immune function, eye health, and cellular balance (Wannasaksri et al., 2021b).

#### 3.4.3 Analgesic, anticholinesterase, neuropharmacological, and antidepressant activities

*Adenia* species showcase significant neuropharmacological potential, offering hope for novel treatments in pain management, anxiety, and depression. *A. trilobata* extracts have demonstrated analgesic effects, with a methanol leaf extract reducing writhing by 57.85% in acetic acid-induced writhing tests and significantly inhibiting formalin-induced licking in mice (Barua et al., 2020). These effects are potentially mediated by flavonoids and terpenoids, which are known for their anti-inflammatory and pain-relieving properties. In addition to pain relief, *A. trilobata* exhibits anxiolytic and antidepressant-like activities. Studies using elevated plus mazes, hole-board tests, forced swimming tests, and tail suspension tests in mice revealed that different fractions of *A. trilobata* extracts could alter behavior, suggesting a modulation of neurotransmitter systems (Aziz et al., 2020). Specifically, open arm entries in the elevated plus maze increased by up to 41.84% with certain extracts, indicating a reduction in anxiety-like behavior (Aziz et al., 2020). In addition, *A. cissampeloides* has demonstrated promising antidepressant and anxiolytic effects. A hydroethanolic extract of *A. cissampeloides* leaves increased swimming activity by 75.20% and climbing activity by 190.00% in the forced swimming test, comparable to the effects of imipramine, a standard antidepressant (Ishola et al., 2015). The extract also showed anxiolytic effects by increasing head dips by 41.67% in the hole-board test and enhancing open-arm entries in the elevated plus maze (Ishola et al., 2015). These effects appear to be linked to the modulation of dopaminergic, adrenergic, and serotonergic pathways, as the antidepressant effects were blocked by sulpiride (a dopamine D2 receptor antagonist) and yohimbine (an α2-adrenoceptor antagonist), while the anxiolytic effects were reversed by cyproheptadine (a 5-HT2 receptor antagonist) (Ishola et al., 2015). Finally, *A. gummifera* has shown anticholinesterase activity, with an IC_50_ value of 0.02 mg/mL, suggesting potential benefits for cognitive function (Adewusi and Steenkamp, 2011). Additionally, steam distillates from *A. gummifera* exhibited anesthetic effects on bees (*Apis mellifera*), likely due to volatile terpenoids interacting with neuronal receptors (Ngarivhume et al., 2008). These diverse neuropharmacological effects suggest that *Adenia* species hold considerable promise for the development of new treatments for pain, anxiety, depression, and cognitive disorders, and warrant further investigation to isolate and characterize the active compounds responsible for these effects.

#### 3.4.4 Antihyperglycaemic and anti-anemic activities

*Adenia* species showcase potential in managing diabetes and anemia and demonstrate diverse antioxidant activity. *A. lobata* stem extracts significantly reduced blood glucose levels in diabetic rats, with the 70% ethanol extract showing up to 82.8% reduction, likely due to flavonoids and phenolics that enhance insulin sensitivity (Sarkodie et al., 2013). Flavonoids’ structure allows them to donate electrons, reducing oxidative stress and promoting iron bioavailability for red blood cell production. Also, *A. lobata* leaf extracts normalized hematological parameters in anemic rats, with the aqueous extract showing a superior recovery rate of 79.66% at 250 mg/kg and increased hemoglobin levels (Florian et al., 2022). Beyond these effects, *A. lobata* demonstrates significant antioxidant properties, reducing malondialdehyde while increasing antioxidant enzyme levels in rats (Fowa et al., 2019) and showing potent DPPH scavenging activity, with the ethyl acetate fraction of the cordate leaf variety exhibiting 63.87% activity (Agoreyo et al., 2012). In addition, *A. gummifera* demonstrated comparable ABTS radical scavenging activity to BHT (Adedapo et al., 2008), *A. trilobata* n-hexane leaf fraction displayed 89.19% scavenging activity (Barua et al., 2020), *A. panduriformis* surpassed ascorbic acid in DPPH quenching (Chibuye et al., 2024), and *A. glauca* compound showed an IC_50_ of 51.56 μg/mL (Immaculate et al., 2022), reinforcing *Adenia*’s potential as a source of therapeutic agents.

#### 3.4.5 Anticoagulant, antithrombotic, and thrombolytic activities

*Adenia* species exhibit promising anticoagulant and antithrombotic properties with insights from a mix of compounds that promote these effects. An *A. cissampeloides* and *Pseudocedrela kotschyi* mixture prolonged aPTT and bleeding time in rats, which are both indicators of anticoagulant activity, with no observed adverse effects below 2000 mg/kg (Nyansah et al., 2016). The same extract also reduced tail infarction and microthrombi in a carrageenan-induced thrombosis model (Nyansah et al., 2021). *A. trilobata* methanol stem extract showed thrombolytic activity, lysing clots by 25.58%, though less effectively than streptokinase, which is a known pharmaceutical agent used to treat blood clots (Barua et al., 2020). Together, the data suggest compounds present in *Adenia* interact with coagulation factors which ultimately disrupt normal blood coagulation processes leading to an increase in bleeding time and reduced clot formation. Furthermore, this body of research highlights several *Adenia* species are demonstrating diverse neuropharmacological activities, particularly in pain management, anxiety, and depression. This is important as it suggests that compounds isolated from the Adenia genus may have a therapeutic effect across multiple targets. For example, *A. trilobata* showed analgesic effects, reducing writhing by up to 57.85% and significantly inhibiting formalin-induced licking, effects that are attributed to flavonoids and terpenoids (Barua et al., 2020). This same extract had demonstrated anxiolytic and antidepressant-like activities, as evidenced by increased open arm entries in animal models of elevated plus maze experiments (up to 41.84%) and altered behavior in forced swimming and tail suspension tests, suggesting neurotransmitter modulation (Aziz et al., 2020). Similarly, *A. cissampeloides* has displayed antidepressant-like activity, increasing swimming activity and climbing activity and anxiolytic effects, increasing head dips and open-arm entries, an effect that is linked to dopaminergic, adrenergic, and serotonergic pathways. Finally, *A. gummifera* displayed anticholinesterase activity and anesthetic effects on bees, a reaction that can be attributed to alkaloids and volatile terpenoids (Adewusi and Steenkamp, 2011; Ngarivhume et al., 2008). The culmination of these results highlights an expansive potential of *Adenia* species to effectively manage pain, anxiety, and depression, which has created a basis for further investigation into these applications.

#### 3.4.6 Anesthetic and uterine contraction properties

The anesthetic properties of steam distillates from the stem of *A. gummifera* were assessed using a diffusion method on *A. mellifera*. Live, direct, and fractional distillates (61°C–80°C) exhibited stronger anesthetic effects, while vacuum distillates were milder. The aqueous stem extract also showed dose-dependent anesthetic effect on African bees, with effectiveness increasing up to a 6.0% concentration, beyond which it plateaued, and excessive exposure proved lethal. Factors such as bee-distillate distance (F2,6 = 5.14; Fcal = 31.0) and container volume (F3,8 = 4.07; Fcal = 66.4) significantly influenced anesthetic activity, indicating that the rate of diffusion of the active components was critical. These components likely include amines and halogenated alkanes. Additionally, the leaf extracts of *A. gummifera* contained gummiferol, a polyacetylene known to be toxic to certain human cancer cell lines (Ngarivhume et al., 2008). The cytotoxicity activity of gummiferol may contribute to the anesthetic effect by disrupting cellular functions in nerve tissues. The SAR of this compound involves its ability to bind to and modulate the activity of neuronal receptors, leading to the observed anesthetic effects. In 2015, Sinei and Mwangi investigated the effects of the aqueous extract of the *A. globosa* tuber on isolated rat uterus preparations and its interaction with prostaglandin F2α and ergometrine, both known uterine stimulants. The study found that the plant extract induced a dose-dependent contraction of the rat uterus. Notably, small doses of prostaglandin F2α (2.5 μg/mL) and ergometrine (0.125 μg/mL) significantly enhanced the contractile effects of the extract, with the presence of prostaglandin F2α increasing the effect (p < 0.02 and p < 0.01). Alone, this dose of prostaglandin F2α resulted in approximately 15% contraction, while ergometrine resulted in approximately 5% contraction on its own (p < 0.05, p < 0.02, and p < 0.01). These findings indicate a synergistic relationship between the extract and these uterine stimulants (Sinei and Mwangi, 2015). The uterine contraction properties of *A. globosa* are likely mediated by the presence of oxytocic compounds, which can stimulate uterine smooth muscle contraction. The synergistic relationship with prostaglandin F2α and ergometrine suggests that the extract may enhance the sensitivity of uterine receptors to these stimulants or act through a similar mechanism (Sinei et al., 1994).

#### 3.4.7 Cytotoxicity activities

The genus *Adenia* has garnered attention in pharmacological research due to its bioactive compounds with significant cytotoxic properties. These include ribosome-inactivating proteins (RIPs), which exhibit promising activities against cancer cells and neurological targets. These properties could be attributed to their strong cell binding, resistance to proteolysis, and efficient endocytosis. These properties enable their retrograde transport along the central nervous system and peripheral nerves, suggesting potential applications in neurophysiology and as components of immunotoxins for targeted cancer therapy and other treatments (Bortolotti et al., 2021). The cytotoxic activity of RIPs is primarily due to their ability to inhibit protein synthesis by depurinating a specific adenine residue in ribosomal RNA, thereby disrupting ribosome function. The structure-activity relationship (SAR) of RIPs is characterized by two key domains: an N-glycosidase domain responsible for the enzymatic activity and a lectin domain that facilitates binding to cell surface glycoproteins or glycolipids, enabling cellular entry (Bortolotti et al., 2021). The specific amino acid sequence and glycosylation patterns of these domains influence their binding affinity and enzymatic activity, ultimately determining their cytotoxicity. For instance, Monti et al. (2007) examined the impacts of lanceolin and stenodactylin, two type 2 RIPs from *A. lanceolata and A. stenodactyla* respectively, on neural cells and their neurotoxicity *in vivo*. The concentrations inhibiting 50% protein synthesis were measured at 10^−11^ M and 10^−12^ M for cerebellar granule neurons, 10^−12^ M and 10^−13^ M for astrocytes, and 10^−13^ M for microglia. Both RIPs were toxic to glial cells, with microglia being the most sensitive, showing 50% cell death at around 10^−14^ M. Stenodactylin demonstrated high neurotoxicity when injected intracerebrally and leading to the loss of cholinergic neurons in the medial septal nucleus following stereotaxic injection of 1.3 ng. The retrograde transport of these RIPs along neurons enables targeted lesioning experiments and offers potential for immunolesioning specific neuronal populations (Monti et al., 2007). The differential cytotoxicity of lanceolin and stenodactylin towards different neural cell types highlights the importance of specific cell surface receptors and endocytic pathways in mediating RIP entry and toxicity. In another study, Stirpe et al. (2007) demonstrated that both RIPs demonstrated the ability to agglutinate red blood cells, inhibit protein synthesis in cell-free systems and whole cells, and depurinate ribosomes and DNA, while not affecting tRNA or poly(A). They were found to be highly toxic, inducing apoptosis in cells and exhibiting LD_50_ values of 8.16 mg/kg for lanceolin and 2.76 mg/kg for stenodactylin in mice after 48 h (Stirpe et al., 2007). The ability of lanceolin and stenodactylin to agglutinate red blood cells and inhibit protein synthesis underscores their potent cytotoxic activity. The lower LD_50_ value of stenodactylin compared to lanceolin indicates that it is more toxic, possibly due to differences in their amino acid sequences, glycosylation patterns, or cellular trafficking. In addition, stenodactylin have been proven to be a promising candidate for anticancer therapy due to its ability to induce apoptosis and necroptosis in treated cells through reactive oxygen species production. In studies involving Raji and Ramos (human Burkitt’s lymphoma) and MOLM-13 (acute myeloid leukemia) cells, stenodactylin demonstrated significant effects on protein synthesis and cell viability. After 48 h of exposure to 10^−9^ M stenodactylin, protein synthesis was nearly completely inhibited across all cell lines, although MOLM-13 cells were the most sensitive, exhibiting an IC_50_ of 3.75 × 10^−12^ M, compared to 1.95 × 10^−11^ M for Raji and 3.49 × 10^−11^ M for Ramos. Cell viability assays indicated that all cell lines showed similar sensitivity to stenodactylin, with EC_50_ values of 2.09 × 10^−10^ M (Raji), 3.43 × 10^−10^ M (Ramos), and 1.06 × 10^−10^ M (MOLM-13). Time-course studies revealed a dose-dependent reduction in MOLM-13 cell viability, with significant effects observed after 12 h and complete loss by 24 h (Mercatelli et al., 2020). Moreover, stenodactylin-induced rapid activation of apoptotic pathways was confirmed through flow cytometry and microscopy, with an IC_50_ of 4 × 10^−14^ M and an EC_50_ of 2.5 × 10^−14^ M noted after 48 h (Polito et al., 2016). The induction of apoptosis and necroptosis by stenodactylin in cancer cells, coupled with its ability to generate reactive oxygen species (ROS), highlights its potential as an anticancer agent. The variations in IC_50_ and EC_50_ values across different cell lines suggest that stenodactylin’s cytotoxicity is influenced by cellular factors such as the expression of specific receptors or the activity of antioxidant defense mechanisms. The rapid activation of apoptotic pathways further supports its potent cytotoxic activity. Furthermore, in 2010, Battelli et al. evaluated the concentration-response curves for protein synthesis and cell viability after 24 h of exposure to RIPs lanceolin, volkensin, and stenodactylin. They found that lanceolin and volkensin exhibited similar cytotoxicities, while stenodactylin was two orders of magnitude more cytotoxic. The IC_50_ values for each RIP closely coincided with the LC_50_ values, although a low percentage of cell viability remained even after complete inhibition of protein synthesis, which occurred at 10^−12^ M for stenodactylin and 10^−10^ M for lanceolin and volkensin. Viability tests indicated that 20% of metabolizing cells persisted after 24 h of treatment at these concentrations. Complete cell death (100%) was observed only after 72 h for both lanceolin and stenodactylin. HeLa cell morphology was assessed by optical microscopy, revealing significant alterations after exposure to 10^−12^ M lanceolin, stenodactylin, or volkensin compared to control cultures (Battelli et al., 2010). The higher cytotoxicity of stenodactylin compared to lanceolin and volkensin reinforces the notion that subtle structural differences among RIPs can significantly impact their biological activity. The persistence of metabolizing cells even after complete inhibition of protein synthesis suggests that cells may activate alternative survival mechanisms, underscoring the complexity of cellular responses to RIP-induced stress. Volkensin, purified from the roots of *A. volkensii*, inhibits protein synthesis in rabbit reticulocyte lysates and HeLa cells. It is highly toxic to mice, with an LD_50_ of 1.38 μg/kg body weight (Barbieri et al., 1984). The potent toxicity of volkensin, as evidenced by its low LD_50_ value, underscores its potential as a cytotoxic agent. Its ability to inhibit protein synthesis in both cell-free systems and whole cells highlights its broad-spectrum activity. Whereas Gummiferol, a type 2 RIP isolated from the leaves of *A. gummifera*, exhibits significant cytotoxic activity against the human epidermoid carcinoma (KB) cell line. The organic portion of a 20% MeOH/CHCl_3_/H_2_O partition from a 50% MeOH/CHCl_3_ extract of the leaves demonstrated an ED_50_ value of 1.1 μg/mL in the kB cell line assay (Fullas et al., 1995; Adedapo et al., 2008). The cytotoxic activity of gummiferol against the kB cell line suggests that it may have potential as an anticancer agent. Its isolation from the organic portion of the extract indicates that it is a relatively non-polar compound, consistent with the properties of many RIPs.

In addition, the cytotoxic effects and the pathways of cell death induced by heterophyllin, a type 2 RIP isolated from *A. heterophylla*, were assessed in three human-derived cell lines: MCF7, NB100, and T24, and compared to ricin (a well-studied type 2 RIP). Heterophyllin was found to entirely eliminate cell viability at nanomolar concentrations, inducing strong apoptosis without necrosis, alongside oxidative stress and necroptosis. Cell viability was assessed after 72 h of incubation using concentration-response experiments. Ricin significantly reduced cell viability starting at 10^−13^ M, whereas heterophyllin began to show significant effects at 10^−12^ M (T24 and MCF7) and 10^−11^ M (NB100). The EC_50_ for heterophyllin was approximately 10^−11^ M across all cell lines. Complete cell killing occurred at 10^−9^ M for T24 and NB100 cells and 10^−10^ M for MCF7 cells. In contrast, ricin demonstrated higher toxicity with an EC_50_ of 10^−13^ M and complete cell killing at 10^−11^ M. Cytofluorimetric analysis using Annexin V-EGFP/Propidium iodide (PI) staining revealed that after 24 h of exposure to 10^−9^ M heterophyllin, over 95% of treated cells were in early or late apoptosis, with minimal necrosis observed. The cytotoxicity of heterophyllin (10^−9^ M) and ricin (10^−11^ M) was evaluated after 24 h, showing that both scavengers and cell death inhibitors significantly increased cell survival (p < 0.0001) in all tested cell lines. In NB100 cells, pre-treatment with scavengers/inhibitors offered greater protection against heterophyllin than ricin. Conversely, in T24 and MCF7 cells, pre-treatment was more effective for ricin than for heterophyllin, particularly in MCF7 cells (Bortolotti et al., 2024). The distinct effects of heterophyllin and ricin on cell viability and the varying degrees of protection conferred by scavengers and cell death inhibitors across different cell lines highlight the complexity of RIP-induced cytotoxicity. The induction of apoptosis without necrosis by heterophyllin suggests a specific mechanism of action distinct from ricin, possibly involving different intracellular targets or signaling pathways. The higher sensitivity of NB100 cells to heterophyllin compared to ricin may be related to differences in cell surface receptors or endocytic pathways, while the opposite trend in T24 and MCF7 cells suggests that these cell lines are more susceptible to ricin’s mechanism of action.

Furthermore, the effects of crude extracts *from A. racemosa, A. fruticosa, A. venenata, A. keramanthus, A. glauca,* and *A. lanceolata* on protein synthesis were assessed using rabbit reticulocyte lysates, HeLa, and Raji cell lines. Hemagglutinating activity was also assessed with normal human erythrocytes (group O, Rh+). The results revealed that, the extracts from *A. racemosa*, *A. fruticosa,* and *A. venenata* exhibited low inhibitory activity, with IC_50_ values ranging from 1.5 to 36 mg/mL. While, extracts from *A. goetzii, A. lanceolata*, and *A. stenodactyla* were highly potent, with IC_50_ values below 1 ng/mL to less than 10 ng/mL. Most extracts, except those from *A. keramanthus*, *A. glauca,* and *A. lanceolata*, demonstrated hemagglutinating activity, particularly strong in *A. goetzii*, *A. ellenbeckii,* and *A. racemosa*. Agglutination was inhibited by 0.2 M galactose in all cases (Pelosi et al., 2005). The varying potencies of crude extracts from different *Adenia* species in inhibiting protein synthesis and inducing hemagglutination reflect differences in their RIP content and lectin profiles. The high inhibitory activity of extracts from *A. goetzii*, *A. lanceolata*, and *A. stenodactyla* suggests that these species are rich in potent RIPs. The hemagglutinating activity of most extracts, particularly those from *A. goetzii*, *A. ellenbeckii*, and *A. racemosa*, indicates the presence of lectins capable of binding to erythrocytes. The inhibition of agglutination by galactose suggests that these lectins recognize galactose-containing glycans on the erythrocyte surface. AHL, a single lectin B chain isolated from *A. hondala* roots, is consider as a human blood group non-specific lectin that agglutinates rabbit erythrocytes. Glycan array analysis revealed that AHL has the greatest affinity for terminal lactosamine or polylactosamine of N-glycans, which are overexpressed in colon cancer and hepatocellular carcinoma. AHL displayed strong binding to human hepatocellular carcinoma HepG2 cells, with a mean fluorescence intensity (MFI) of 59.1, which was effectively blocked by 93.1% by asialofetuin. It also exhibited dose- and time-dependent growth inhibitory effects on HepG2 cells, with an IC_50_ of 4.8 μg/mL at 72 h. Specifically, AHL (at 10 μg/mL) inhibited HepG2 cell growth by 43% (n = 6, P < 0.01) at 48 h and 76% (n = 6, P < 0.001) at 72 h (Sharma et al., 2018). Additionally, in 2021 Inamdar et al. assessed the interaction of AHL with human colon cancer epithelial HT-29 cells and colon cancer tissues. For that, the cell viability was assessed using the MTT assay, while cell surface binding, apoptosis, and ROS production were analysed by flow cytometry with the Annexin-V-PI and DCFDA kits. Immunohistochemistry utilised biotinylated AHL, and protein purification was conducted via affinity chromatography with asialofetuin-coupled Sepharose-4B. Their results showed that, AHL demonstrated strong binding to HT-29 cells, with a Mean Fluorescence Intensity of 12.4, which was inhibited by asialofetuin. It reduced HT-29 cell growth in a dose- and time-dependent manner, yielding an IC_50_ of 2.5 μg/mL, and showed differential binding to normal and cancerous human tissues. AHL induced apoptosis, with early apoptotic populations increasing to 25.1% and 36% at 24 h and 48 h, respectively, and necrotic populations at 1.5% and 4.6%. Moreover, AHL triggered the release of Reactive Oxygen Species in a dose-dependent manner (Inamdar et al., 2021). The preferential binding of AHL to lactosamine or polylactosamine of N-glycans, which are overexpressed in colon cancer and hepatocellular carcinoma, suggests that it may have potential as a targeted therapeutic agent for these cancers. The dose- and time-dependent growth inhibitory effects of AHL on HepG2 and HT-29 cells, coupled with its ability to induce apoptosis and ROS production, further support its potential as an anticancer agent. The differential binding of AHL to normal and cancerous human tissues suggests that it can selectively target cancer cells while sparing normal cells, reducing the risk of toxicity.

### 3.5 Toxicity and safety

The toxicity of *A. cissampeloides* has been extensively studied, particularly regarding its effects on catfish larvae and juveniles. The methanol extract of the stem was tested under static bioassay conditions for 96 h, revealing various pathological changes in fish larvae, including erratic swimming, moribund behavior, depigmentation, and mortality at concentrations of 25, 50, 75, and 100 mg/L. The lethal concentration for 50% mortality (LC_50_) was determined to be 50 mg/L within 72 h, indicating high toxicity to catfish juveniles (Agbor and Okoi, 2014). In a separate study, Ibiam et al. (2015) evaluated the acute toxicity of stem-bark aqueous extract of *A. cissampeloides* in *Clarias batrachus* juveniles. The study involved 144 fish randomized into groups exposed to varying concentrations (0.0, 1.25, 2.50, 5.0, 10.0, and 20.0 g/L) for 72 h. The calculated LC_50_ values were 5.0 g/L at 24 h and 2.5 g/L at both 48 and 72 h, demonstrating a time and concentration-dependent toxic effect. Significant (P > 0.05) increases in aspartate aminotransferase (AST) activity and other biochemical parameters were noted, highlighting the acute toxicity of the extract (Ibiam et al., 2015). Further research by Ekpo et al. (2017) assessed the acute toxicity of the leaf 70% ethanol extract of *A. cissampeloides* on early developmental stages of farmed African catfish, with 160 fingerlings exposed to concentrations of 25, 50, and 100 mg/L for 24–96 h. The study found a significant increase in mortality that was dependent on the dose, with an LC_50_ of 42.38 mg/L for 96 h, indicating acute toxic effects on the fish (Ekpo et al., 2017). Another study involving 370 catfish (*Clarias gariepinus*) examined the effects of varying concentrations (0, 25, 50, 75, 100 mg/L) of the leaf extract of *A. cissampeloides* over different durations (24, 48, 72, and 96 h). Results showed that both concentration and exposure duration significantly (p < 0.05) affected mortality rates, with the highest mortality observed at the highest concentration and longest exposure time (Uno et al., 2018). The toxicity of *A. cissampeloides* in fish is likely due to the presence of toxic compounds such as saponins, alkaloids, and cyanogenic glycosides. Saponins can disrupt cell membranes, leading to hemolysis and tissue damage, while alkaloids can interfere with neuronal function and cause paralysis. Cyanogenic glycosides release cyanide upon hydrolysis, inhibiting cellular respiration and causing asphyxiation. The SAR of these compounds involves their ability to interact with cellular targets, disrupting normal physiological processes and leading to toxicity. The concentration- and time-dependent effects observed in these studies underscore the importance of dose and duration of exposure in determining the severity of toxicity. In contrast, a study evaluated the therapeutic and potential toxic effects of an aqueous extract of *A. cissampeloides* in seven female hypertensive subjects undergoing treatment and seven matched controls who had not yet started the treatment. Diastolic and systolic blood pressures were measured, and serum levels of various enzymes, including creatine kinase (CK), α-hydroxybutyrate dehydrogenase (HBDH), lactate dehydrogenase (LDH), and aspartate aminotransferase (AST), were analysed to assess the extract’s effectiveness as an antihypertensive agent. The results showed that systolic blood pressure was significantly reduced in subjects taking *A. cissampeloides* compared to controls (p < 0.01). Although CK and HBDH levels were reduced in the treatment group, these differences did not reach statistical significance. Additionally, conjugated and total bilirubin levels were significantly reduced in the subjects on *A. cissampeloides* (p < 0.05). The findings suggest that the plant preparation does not exhibit hepatotoxic or nephrotoxic effects (Nyarko and Addy, 1990). The lack of hepatotoxic or nephrotoxic effects observed in hypertensive subjects treated with *A. cissampeloides* aqueous extract suggests that the toxic compounds present in the plant may be poorly absorbed or rapidly metabolized, or that the concentration of these compounds in the aqueous extract is below the threshold for toxicity. The reduction in systolic blood pressure may be due to the presence of compounds that act as vasodilators or ACE inhibitors. However, it is important to note that this study involved a small sample size and further research is needed to confirm these findings and to assess the long-term safety of *A. cissampeloides* in humans. In 2020, Barua et al. investigated the lethality of the *A. trilobata* stems and leaves methanol extracts, as well as their n-hexane fractions, using the brine shrimp (*Artemia salina*) bioassay. They found that none of the extracts demonstrated toxicity when compared to vincristine sulfate (2.16 μg/mL), the positive control. The results indicated that the methanol stem extract had a moderately toxic effect, with an LC_50_ of 328 μg/mL, while the n-hexane leaf fraction exhibited weak toxicity, with an LC_50_ of 616.85 μg/mL (Barua et al., 2020). The moderate toxicity of the methanol stem extract and the weak toxicity of the n-hexane leaf fraction from *A. trilobata* in the brine shrimp bioassay suggest that these extracts contain relatively low concentrations of toxic compounds. The differences in toxicity between the methanol and n-hexane extracts may be due to the presence of different classes of compounds with varying degrees of toxicity.

Volkensin, a type 2 RIP derived from the roots of *A. volkensii*, is a highly toxic protein with a LD_50_ for rats reported to be between 50 and 60 ng/kg. It is closely related to other well-known toxins such as abrin and ricin, which have been recognized since the late 20th century (Stirpe et al., 1985; Chambery et al., 2004; Chambery et al., 2007; Severino et al., 2009). In experimental settings, the toxicity of volkensin has been evaluated in male Swiss mice, where it was administered intraperitoneally at varying doses (0.1–10 µg per 100 g body weight) dissolved in saline. This method of administration allows for the assessment of its lethal effects in a controlled environment (Barbieri et al., 1984). In addition, modeccin, another toxin purified from the roots of *A. digitata*, has been studied for its effects on protein synthesis and toxicity in animal models. Two isoforms, modeccin 4B and modeccin 6B, were isolated, with modeccin 6B exhibiting slightly less toxicity than modeccin 4B. Notably, modeccin 6B does not agglutinate erythrocytes and is a more effective inhibitor of protein synthesis in rabbit reticulocyte lysates, achieving 50% inhibition at a concentration of 0.31 μg/mL. While the LD_50_ of modeccin 6B was not explicitly determined, experiments showed that all rats administered 1 µg of the toxin per 100 g body weight died, whereas those receiving 0.1 µg survived, indicating its intermediate toxicity compared to modeccin 4B and its reduced form treated with 2-mercaptoethanol (Barbieri et al., 1980). Another study demonstrated that modeccin inhibits protein synthesis both *in vitro* in rabbit reticulocyte lysates and Ehrlich ascites cells, with the inhibitory effect diminished in the presence of lactose. Interestingly, dissociating modeccin into subunits reduces its toxicity *in vivo*, as seen in rats that survived doses of 5 µg of dissociated modeccin per 100 g body weight. Furthermore, studies revealed that modeccin is generally more toxic to rats than to mice, causing pathological changes somewhat similar to those observed in ricin poisoning (Gasperi-campani et al., 1978). Furthermore, studies have demonstrated that stenodactylin, a type 2 RIP derived from the *A. stenodactyla* caudex, exhibits high toxicity in mice, resulting in 100% mortality among those injected with 1.21 μg/kg within 7 days (Tosi et al., 2009). The high toxicity of volkensin, modeccin, and stenodactylin is due to their ability to inhibit protein synthesis, a critical cellular function.

Importantly, a case was reported involving four siblings who unintentionally ingested the *A. ellenbeckii* fruits in the rural Togdheer region of northern Somaliland. Following consumption, they experienced severe illness, including sweating, fever, frequent vomiting, and itching. One sibling progressed to acute kidney injury, resulting in a fatal outcome (Rage et al., 2024). In South Africa, the genus has garnered attention primarily due to poisoning incidents associated with *A. digitata*. In 1922, a poisoning event resulted in the death of an adult who mistakenly chewed the tubers of *A. digitata*, believing them to be from a cucurbitaceous plant. Previous reports indicated cases of poisoning among children, including a death attributed to this species in 1928. Investigations at the Veterinary Research Laboratory in Onderstepoort identified two toxic principles: hydrocyanic acid and modeccin. Hydrocyanic acid was found in the fresh leaves of both *A. digitata* and *A. glauca*, although the latter’s root was deemed edible. Additionally, observations were made of children consuming the fruits of *A. glauca*, which they described as pleasant. Confusion arises regarding the consumption of the sap from the “tuber” of *A. midtiflora*, likely due to its overlap with the non-toxic and edible *A. glauca*. Furthermore, *A. venenata* and *A. palmata* are recognized as having poisonous tubers (Liebenberg, 1935). These case reports and historical accounts of poisoning incidents highlight the potential dangers associated with the ingestion of certain *Adenia* species. The presence of hydrocyanic acid (cyanide) in *A. digitata* and *A. glauca* can lead to cyanide poisoning, which inhibits cellular respiration and causes rapid death. Modeccin, another toxic compound found in *A. digitata*, inhibits protein synthesis and can cause organ damage. The confusion between toxic and non-toxic species underscores the importance of accurate identification and caution when using *Adenia* plants for medicinal or food purposes. The symptoms reported in the siblings who ingested *A. ellenbeckii* fruits, including acute kidney injury, suggest that the toxic compounds present in this species can cause severe organ damage.

## 4 Conclusion

In conclusion, the genus *Adenia* represents a significant area of study at the intersection of traditional medicine and modern pharmacology. While many species within this genus are recognized for their ethnobotanical uses, particularly in treating conditions like hypertension and infections, the presence of toxic compounds—especially in species like *A. digitata* and *A. volkensii*—raises important safety concerns. Phytochemical analyses have identified various bioactive compounds, such as ribosome-inactivating proteins, alkaloids, flavonoids, and glycosides, which contribute to their therapeutic effects, including anticancer, antimicrobial, antioxidant, and analgesic properties. However, the variability in toxicity among different species and the influence of factors such as geographical location and preparation methods necessitate careful evaluation. Future research should prioritize standardizing methodologies for assessing both the safety and efficacy of *Adenia* species, alongside comprehensive toxicological studies to elucidate their mechanisms of action. By addressing these safety concerns, the therapeutic potential of the genus *Adenia* can be more effectively harnessed in modern medical practices.
