# Supplementary material for: Review of the ethnobotany, phytochemistry, pharmacology, and toxicity studies of the genus Adenia
Source: Front Pharmacol. 2025 May 21;16:1581659. doi: 10.3389/fphar.2025.1581659 (PMC12135688; doi:10.3389/fphar.2025.1581659)
Supplement: Supplementary file 1 [file Supplementaryfile1.pdf]

SUPPLEMENTARY MATERIAL TO

Review of the Ethnobotany, Phytochemistry, Pharmacology, and Toxicity Studies of the Genus *Adenia*.

Iliassou L. Mouafon* and David R. Katerere

Department of Pharmaceutical Sciences, Tshwane University of Technology, Pretoria 0083, South Africa

*Corresponding authors:

Tel.: +27 072 366 1807; e-mail address: mouafon-lahi@tut.ac.za / lahiliass@yahoo.fr (I.L. Mouafon)

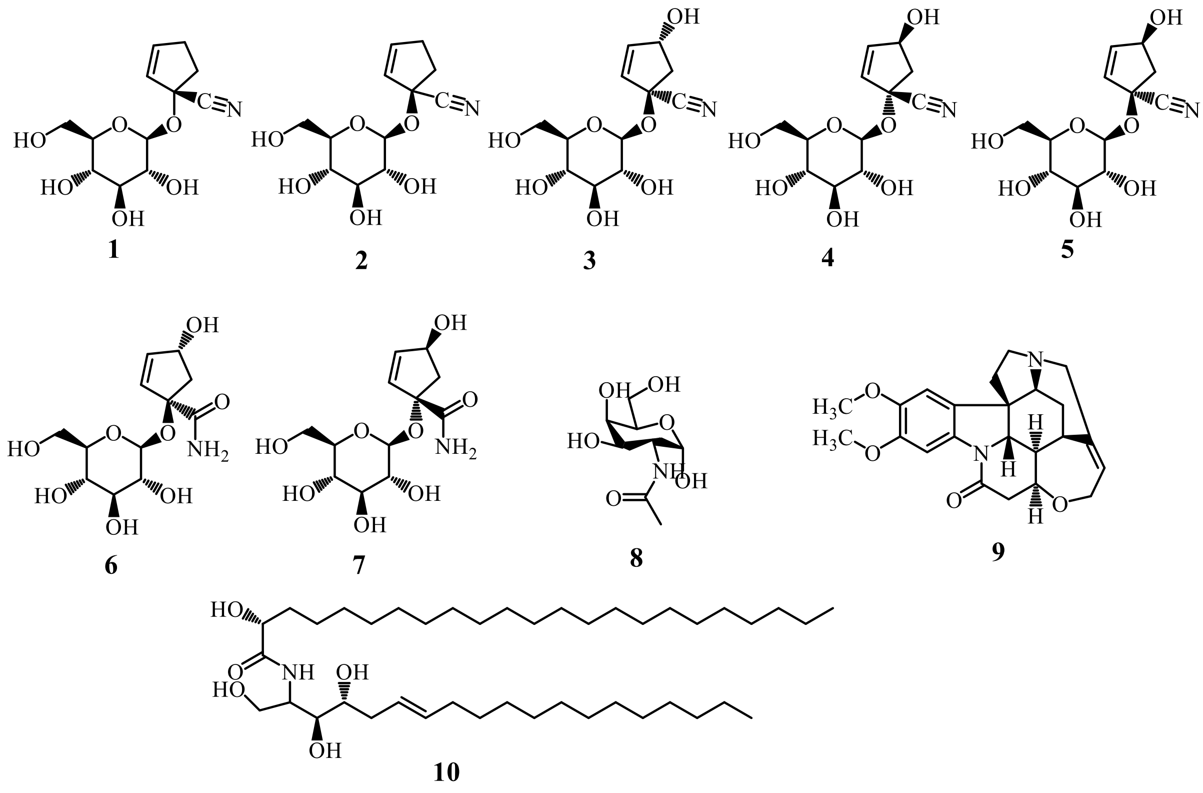

Figure 2. Cyanohydrin glycosides (1-8), Alkaloid (9), and Ceramide (10) from *Adenia* species.

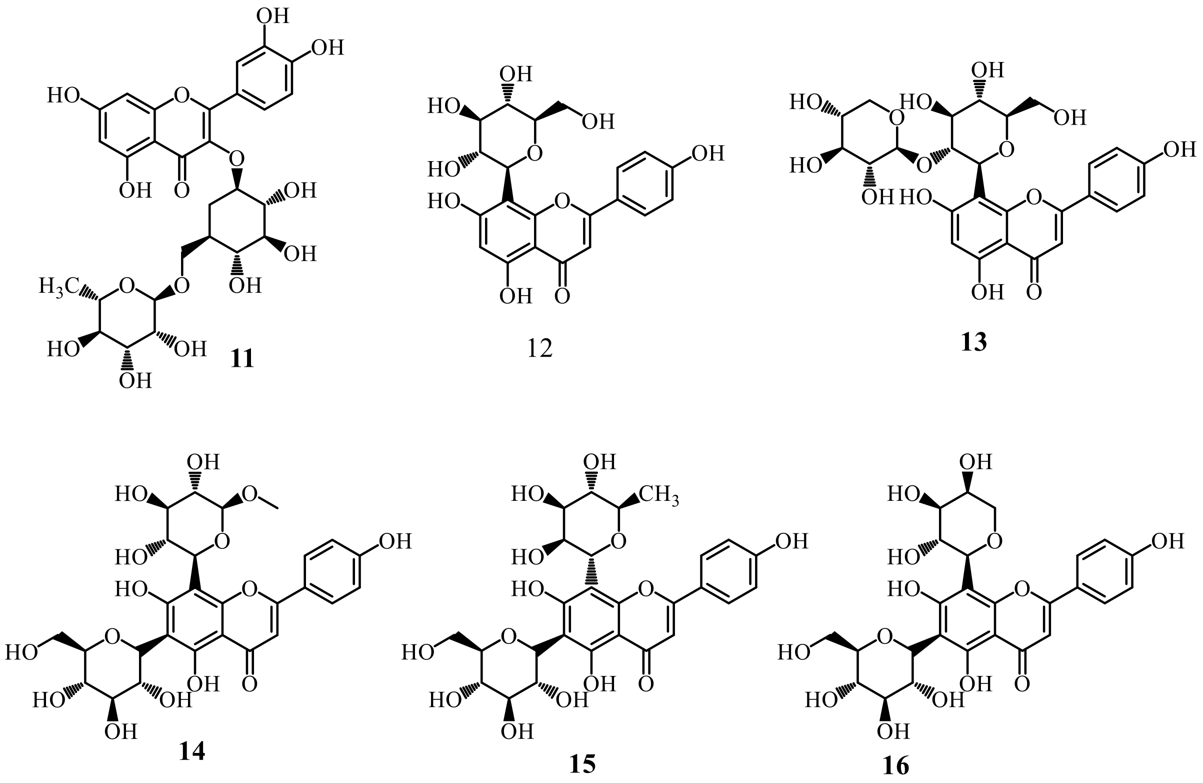

Figure 3. Glycosyl flavonoids from *Adenia* species.

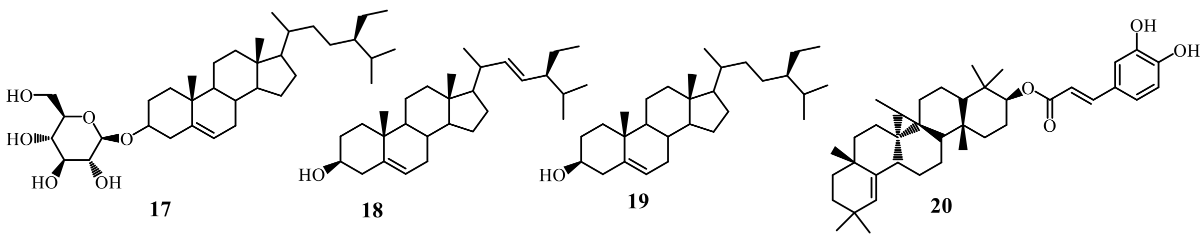

Figure 4. Steroids and Triterpenes from *Adenia* species.

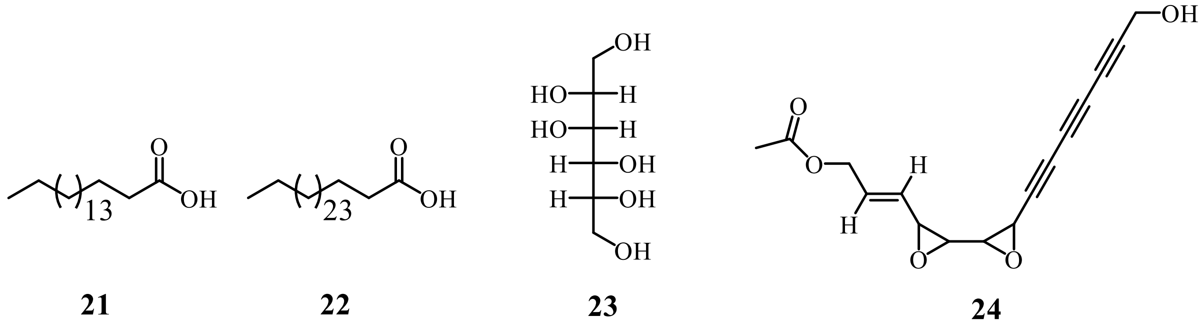

Figure 5. Fatty Acid and polyacetylene from *Adenia* species.

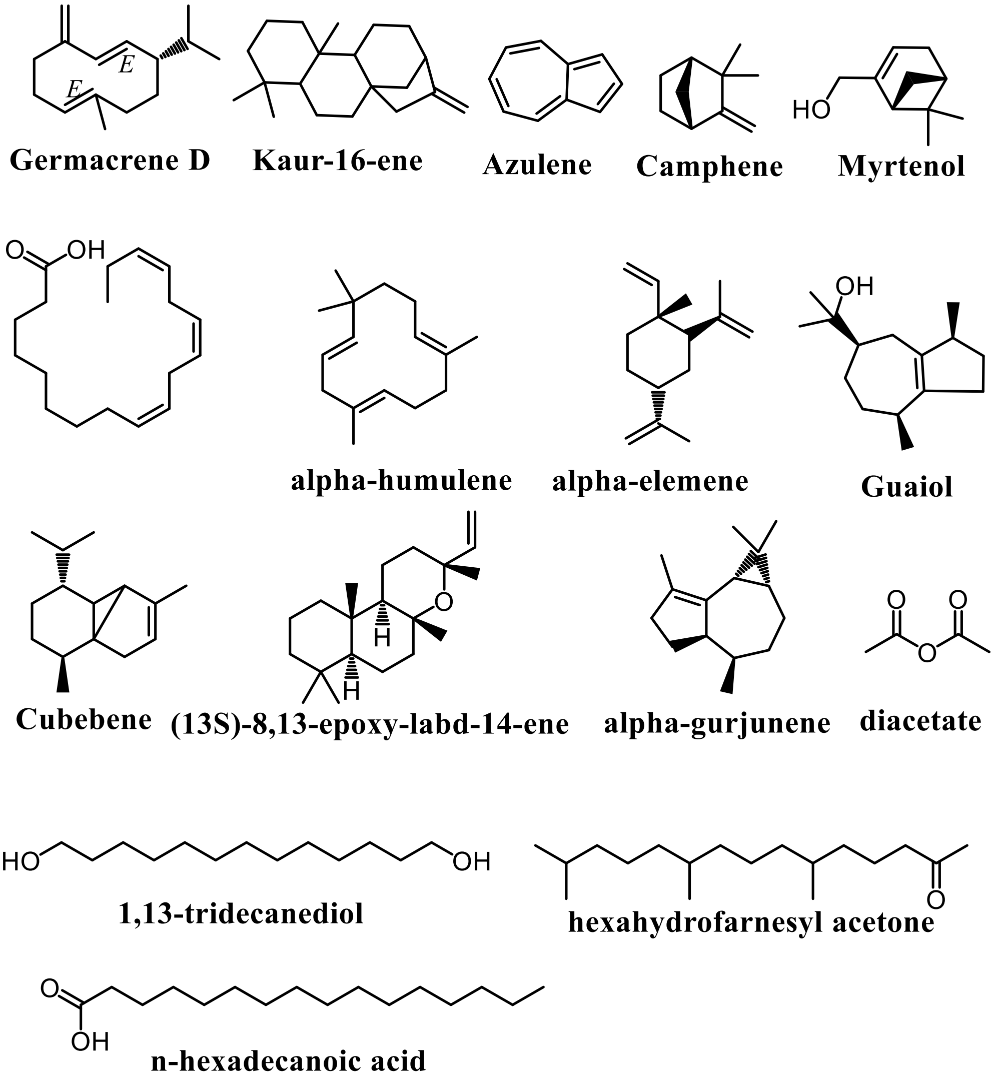

Figure 6. Main volatile chemical constituents found in *Adenia* species.
